# The Effects of Colostrum Bovinum Supplementation on Human Body Fat Content and/or Blood Lipid Profile: A Systematic Review of Clinical Trials

**DOI:** 10.3390/nu18101579

**Published:** 2026-05-15

**Authors:** Zuzanna Goluch, Ewelina Książek, Aldona Wierzbicka-Rucińska, Ireneusz Skawina, Robert Dudkowiak

**Affiliations:** 1 Department of Food Technology and Nutrition, Wroclaw University of Economics and Business, Komandorska 118/120, 53-345 Wrocław, Poland; 2 Department of Agricultural Engineering and Quality Analysis, Wrocław University of Economics and Business, Komandorska 118/120, 53-345 Wrocław, Poland; ewelina.ksiazek@ue.wroc.pl; 3 Department of Clinical Biochemistry, Radioimmunology and Experimental Medicine, Children’s Memorial Health Institute, 04-730 Warsaw, Poland; a.wierzbicka-rucinska@ipczd.pl; 4 District Sanitary and Epidemiological Station, Wałbrzyska 15, 58-100 Świdnica, Poland; ireneusz_skawina@o2.pl; 5 Department of Gastroenterology and Internal Medicine, University Clinical Center of Medical University of Warsaw, Banacha 1a, 02-097 Warsaw, Poland; r.dudkowiak@szpital-marciniak.wroclaw.pl; 6 Endoscopy Department, T. Marciniak Lower Silesian Specialist Hospital—Emergency Medicine Center, Gen. August Emil Fieldorfa 2, 54-049 Wrocław, Poland

**Keywords:** colostrum, dietary supplements, body fat, lipid profiles, cholesterol, triacylglycerols, PRISMA

## Abstract

*Bovine colostrum* (COL) is widely used in dietary supplements, and previous studies have suggested its potential benefits for immune function, selected clinical conditions, wound healing, and athletic performance. This systematic review analyzed clinical trials published between 2001 and 2025 that investigated the effects of COL on human body fat and blood lipid profiles. The review was conducted in accordance with PRISMA guidelines, and study quality was assessed using Cochrane risk-of-bias tools. Thirteen studies were included. One study in older adults reported that COL supplementation at 60 g/day for 8 weeks significantly reduced body fat percentage by 0.4% (*p* < 0.05). Another study found that COL supplementation at 10 g/day combined with plant proteins for 12 weeks significantly attenuated the increase in leg tissue fat percentage compared with placebo (PLA) (0.48 ± 1.29% vs. 1.12 ± 1.27%, respectively; *p* < 0.05). Changes in blood lipid profiles were reported in two studies. In individuals with type 2 diabetes, COL supplementation at 10 g/day for 4 weeks significantly reduced total cholesterol (TC) and triglyceride levels in both men and women, by 8.27% vs. 7.62% and 11.96% vs. 21.46%, respectively. In another study involving older adults, COL supplementation at 30 g/day for 12 weeks significantly reduced TC (5.88 to 5.38 mmol/L) and low-density lipoprotein cholesterol (LDL-C) (3.68 to 3.28 mmol/L) compared with PLA. Owing to substantial methodological heterogeneity and inconsistent findings, further randomized, double-blind trials are needed in larger groups of overweight or obese participants, with intervention periods lasting at least six months. Future studies should use a standardized COL dose of 20–25 g/day, controlled caloric deficits, and a four-arm design comparing placebo and COL under normocaloric and energy-restricted dietary conditions. Assessments should include blood metabolic biomarkers, body composition measured by dual-energy X-ray absorptiometry, gut microbiome composition, and fecal short-chain fatty acids to determine whether any observed benefits are attributable to COL alone or to its combination with dietary intervention.

## 1. Introduction

Colostrum, also referred to as “early milk,” is a secretion of the mammary glands of all mammals, including humans, produced toward the end of pregnancy and during the first several dozen hours postpartum, usually up to 48–72 h after delivery. Its primary functions are to provide the newborn with essential nutrients required for growth and development, to confer passive immunity—although transient, it is critical during the first weeks of life through the transfer of maternal antibodies to the offspring—and to stimulate maturation of the gastrointestinal tract [1,2,3,4].

Colostrum has a unique and species-specific composition. Human colostrum provides the newborn with high concentrations of immunoglobulins (mainly sIgA, IgG, IgM, IgD, and IgE), antimicrobial proteins and peptides (lactoferrin, lysozyme, lactoperoxidase, and defensins), as well as viable stem cells and leukocytes that perform protective functions. Colostrum contains human milk oligosaccharides (HMOs), which act as prebiotics and support microbiome development [5,6].

A recently published review [7] highlighted the bioactive growth factors present in colostrum, including insulin-like growth factors (IGF-1 and IGF-2), transforming growth factors (TGF-α, TGF-β1, and TGF-β2), epidermal growth factor (EGF), and platelet-derived growth factor (PDGF). These factors may exert beneficial effects in humans by jointly regulating cell proliferation, differentiation, and survival, and their biological activities have been partially confirmed in experimental and clinical studies in humans and animal models. Their reported effects include accelerated wound healing, for example through EGF- and TGF-mediated stimulation of epithelial regeneration; support for tissue regeneration and angiogenesis; promotion of muscle mass and bone mineral density via activation of the growth hormone/IGF-1 axis, leading to increased protein synthesis, muscle strength, and recovery; improvement of intestinal barrier integrity through stimulation of epithelial repair and reduction in gastrointestinal inflammation; modulation of immune responses, particularly through the anti-inflammatory effects of TGF-β and its role in strengthening the intestinal barrier; beneficial effects on skin cells, including keratinocyte proliferation, protection against oxidative and UV-induced stress, and improved skin barrier integrity; and improvement of glucose metabolism and insulin sensitivity, which may support healthy body weight maintenance and metabolic function. Furthermore, owing to their immunomodulatory properties, these components may have the potential to alleviate symptoms of autoimmune diseases and support immune system development, acting synergistically with the immunoglobulins and lactoferrin present in colostrum. However, large randomized, placebo-controlled trials in humans are still lacking. Such studies are needed to clearly confirm the mechanisms of action of growth factors, determine optimal dosing, and assess the long-term safety of orally administered growth factors derived from bovine colostrum.

In addition, colostrum contains vitamins (A, D, E, K, B1, B2, B6, B12, and C) and minerals (Ca, P, Mg, K, Na, Fe, Zn, Se, and Cu). From a nutritional perspective, human colostrum is characterized by a markedly higher content of protein, growth factors, cytokines, and other bioactive compounds than mature milk, while containing lower amounts of fat and lactose, which makes it optimally suited to the infant’s physiological needs [5,8]. In recent years, several literature reviews have addressed the importance of human colostrum in infant feeding and its contribution to appropriate growth and health outcomes [6,8,9,10].

However, colostrum obtained from various animal species, including goats, cows, sheep, and camels, is currently used in the global production of dietary supplements [11]. Compared with human colostrum, the colostrum of these animal species differs somewhat in composition and biological properties [10,11,12,13,14,15], largely owing to the environmental conditions in which these animals live and their physiological adaptation to nourishing their own offspring. For example, these types of colostrum differ in their content of energy (Human < Bovine < Caprine < Camel < Ovine), protein (Human < Caprine < Bovine < Camel < Ovine), fat (Human < Bovine < Caprine < Camel < Ovine), lactose (Ovine < Camel < Bovine < Caprine < Human), IgG (Human < Caprine < Bovine < Camel < Ovine), lactoferrin (Bovine < Caprine < Ovine < Human < Camel), oligosaccharides (Bovine < Ovine < Caprine < Camel < Human), and leptin (Bovine < Caprine < Ovine < Human < Camel) [12,13,14,15,16,17,18,19,20]. Although supplements produced from ovine, caprine, or camel colostrum remain niche products, they offer an alternative for individuals with allergies or those seeking specific bioactive profiles. Nevertheless, bovine colostrum is of greatest relevance in human nutrition—including in infants, children, adults, older individuals, and physically active populations—and is the most widely available on the market in the form of dietary supplements (liquid formulations, hard capsules, powder sachets, chewing gums, lozenges, gummies, and bars), as well as functional foods and nutraceuticals [16,21].

The global colostrum market was valued at USD 386.71 million in 2025 and is projected to reach USD 513.17 million by 2033, with a compound annual growth rate (CAGR) of 3.60% over the forecast period. This growth is largely attributable to the increasing popularity of colostrum-based supplements in sports nutrition and immune health support [22].

Numerous literature reviews have summarized previous studies on the potentially beneficial effects of bovine colostrum supplementation on immune function, gastrointestinal, cardiovascular, respiratory, and endocrine disorders, metabolic and autoimmune diseases, wound regeneration and healing, and athletic performance [23,24,25,26]. However, no systematic review has been identified that specifically addresses its effects on body fat content and blood lipid profiles.

Therefore, the primary aim of this paper is to provide a systematic review of clinical studies investigating the effects of dietary *bovine colostrum* supplementation on body fat content and blood lipid profiles. Through the rigorous selection and evaluation of relevant evidence, this review also illustrates how approaches to colostrum supplementation and its associated health effects have evolved over the past 25 years.

## 2. Materials and Methods

This systematic review was reported in accordance with the Preferred Reporting Items for Systematic Reviews and Meta-Analyses (PRISMA) guidelines [27].

The literature search for this systematic review was restricted to clinical trials conducted in adult human populations over the past 25 years (2001–2025). Six databases were selected for the literature search: EBSCO, Embase, Cochrane Library, PubMed, Scopus, and Web of Science. The search and study selection process was performed independently and in parallel by three reviewers.

The search strategy combined MeSH terms and free-text keywords, using quotation marks, field tags, and Boolean operators. Because the available query options differed across databases, including the possibility of filtering by study design (e.g., clinical trial), database-specific search stages and phrases were developed. Two search queries were applied in each database. The first query comprised the following elements: (1) the keywords Colostrum AND (Lipoprotein OR lipid OR Triglyceride* OR Triacylglycerol* OR Cholesterol OR HDL OR LDL OR apolipoprotein* OR “lipid profile”)* AND trial; (2) publication years 2001–2025; (3) publication type: article; (4) language: English; and (5) source type: journal. The second query included: (1) the keywords Colostrum AND (“body fat” OR “body composition” OR “tissue fat” OR “adipose fat”) AND trial; (2) publication years 2001–2025; (3) publication type: article; (4) language: English; and (5) source type: journal.

For databases that are not exclusively biomedical, screening was conducted across the following fields: title, abstract, and keywords (Scopus—Core Collection); topic (Web of Science—MEDLINE); and all fields in EBSCO. The results retrieved from each database were exported as CSV or Excel files, and a summary was prepared for each source, including bibliographic details and abstracts. The result files have been deposited in the Repository for Open Data: (https://doi.org/10.18150/KF1IKG).

**Figure 1:**
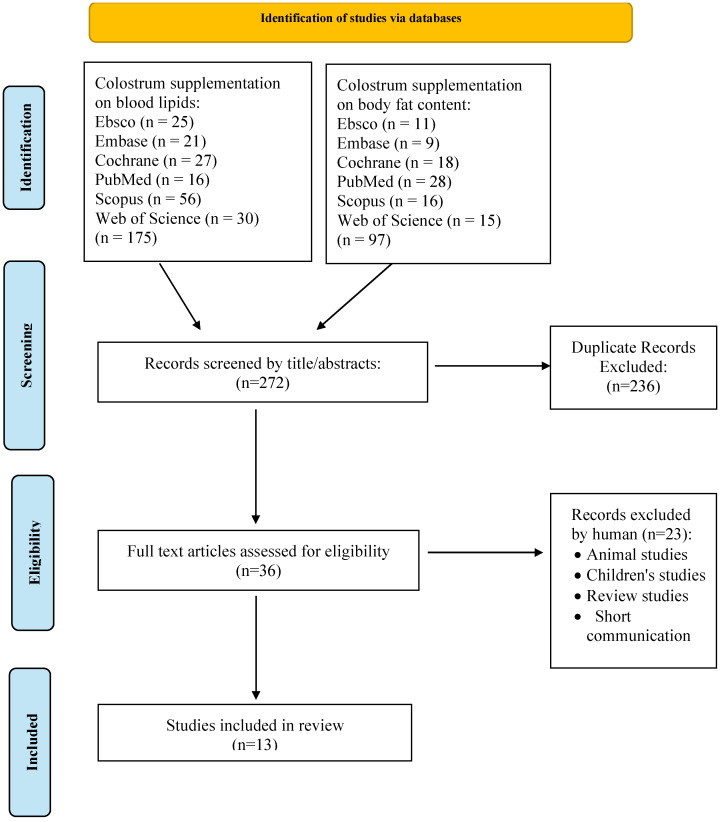
PRISMA flow diagram representing the screening strategy and selection process for research articles. Process of selecting eligible studies.

The initial search using the two queries yielded 272 records. After removing 236 duplicates, 36 records remained. Of these, 22 studies were excluded after discussion, including animal studies, pediatric studies, review articles, and short communications. One clinical trial (ISRCTN15330940 https://www.isrctn.com/ISRCTN15330940 accessed on 12 May 2026) had already been published as a full article [28], which was therefore included in the analysis. Ultimately, 13 full-text articles were included in the review, including one article identified in both search queries [29]. The literature search process is presented in Figure 1.

The methodological quality of the included studies was evaluated by assessing risk of bias according to the guidance in the Cochrane Handbook for Systematic Reviews of Interventions [30]. Randomized controlled trials were assessed using the revised Cochrane risk-of-bias tool for randomized trials (RoB 2), whereas non-randomized studies of interventions were evaluated using the ROBINS-I (Risk Of Bias In Non-randomized Studies of Interventions) tool. For crossover trials, the RoB 2 version adapted for crossover designs was applied. The assessment process was based on the signaling questions specified for each instrument, followed by domain-level and overall judgments derived according to the recommended decision algorithms.

For randomized trials, five RoB 2 domains were assessed and are presented in the tables as D1–D5: D1, bias arising from the randomization process; D2, bias due to deviations from intended interventions; D3, bias due to missing outcome data; D4, bias in measurement of the outcome; and D5, bias in selection of the reported result. In crossover trials, the assessment of bias arising from the randomization process additionally considered issues specific to this design, including the balance of intervention sequences, the adequacy of the washout period, and the likelihood of carryover effects.

For non-randomized studies, ROBINS-I was used to evaluate seven domains, presented in the tables as D1–D7: D1, bias due to confounding; D2, bias in selection of participants into the study; D3, bias in classification of interventions; D4, bias due to deviations from intended interventions; D5, bias due to missing data; D6, bias in measurement of outcomes; and D7, bias in selection of the reported result.

Within the RoB 2 framework, each domain was categorized as low risk of bias, some concerns, or high risk of bias. For ROBINS-I, the standard categories of low, moderate, serious, or critical risk of bias were used. To facilitate consistent interpretation across study designs within the evidence synthesis, a conservative harmonization approach was adopted. Accordingly, ROBINS-I judgments of moderate, serious, and critical risk of bias were interpreted as reflecting an increased risk of bias, whereas low risk of bias was considered directly comparable to the corresponding RoB 2 category.

Overall judgments were assigned according to the recommendations specific to each tool. For RoB 2, the overall risk-of-bias judgment reflected the highest level of concern identified across the assessed domains. For ROBINS-I, the overall judgment was determined in accordance with the tool’s interpretative guidance, with particular caution exercised when key methodological details were missing, insufficiently reported, or unclear.

Risk-of-bias assessments were performed independently, and any disagreements were resolved by discussion and re-examination of the full-text articles until consensus was reached. When methodological reporting was incomplete, a judgment of some concerns was assigned for the relevant RoB 2 domain, or a cautious judgment was applied under ROBINS-I. When insufficient reporting was considered likely to compromise the credibility of the findings or suggested a plausible risk of systematic distortion of the estimated effect, the study was judged as having high risk of bias or the corresponding elevated ROBINS-I level.

The certainty of evidence for the main outcomes was assessed using the Grading of Recommendations Assessment, Development and Evaluation (GRADE) approach, in accordance with the recommendations of the GRADE Handbook. The Summary of Findings (SoF) table was prepared following the Cochrane/GRADE framework to present the main outcomes, the number of participants and studies, the direction of effect, and the certainty of evidence.

The outcomes selected for GRADE assessment were aligned with the aim of this systematic review and included body fat/fat mass or percentage body fat, regional fat mass, total cholesterol (TC), low-density lipoprotein cholesterol (LDL-C), high-density lipoprotein cholesterol (HDL-C), triglycerides (TG), and body weight/body mass index (BMI) as secondary anthropometric outcomes.

The certainty of evidence was assessed separately for each outcome across five GRADE domains: risk of bias, inconsistency, indirectness, imprecision, and publication bias. Randomized controlled trials were initially considered high-certainty evidence and were downgraded when serious or very serious concerns were identified. Non-randomized, open-label, or uncontrolled intervention studies were interpreted with caution due to their inherently increased susceptibility to confounding and bias.

Evidence was downgraded for risk of bias when studies showed incomplete reporting of randomization or allocation concealment, relevant attrition, crossover-specific limitations, open-label design, selective reporting concerns, or the use of multi-ingredient interventions that limited attribution of effects to bovine colostrum itself. Evidence was downgraded for inconsistency when findings differed across studies or when beneficial effects were observed only in isolated trials. Evidence was downgraded for indirectness when populations, intervention protocols, doses, comparators, co-interventions, or outcome assessment methods differed substantially from the review question. Evidence was downgraded for imprecision when the number of participants or studies per outcome was limited. Publication bias was considered suspected when the number of studies was small and when interventions were industry supported or supplement provided.

The final certainty of evidence for each outcome was categorized as high, moderate, low, or very low, in accordance with the GRADE approach.

A meta-analysis was not performed because the included studies showed substantial clinical and methodological heterogeneity. Although some outcomes, such as LDL-C, were reported across multiple studies, the available data were insufficiently homogeneous for quantitative synthesis. Studies differed in design (randomized controlled trials, crossover trials, and open-label or single-arm interventions), in participant characteristics (healthy adults, older adults, and patients with metabolic diseases), in the form and dose of bovine colostrum supplementation, and in the type of comparator used. In addition, several studies used multi-ingredient preparations, making it difficult to isolate the independent effect of bovine colostrum. For some outcomes, data were reported only as within-group changes, percentage changes, or responder analyses, without complete between-group estimates and measures of variance. Therefore, pooling these data would not provide a reliable estimate of the intervention effect and could lead to misleading conclusions. Consequently, a qualitative synthesis was performed, supported by a formal GRADE assessment of the certainty of evidence.

## 3. Results

### 3.1. Risk-of-Bias Assessment

The results of the risk-of-bias assessment are presented in Table 1 and Table 2. Overall, the methodological quality of the included studies was heterogeneous. Among randomized controlled trials, most studies were judged to have either some concerns or a high risk of bias, whereas all non-randomized studies were judged to be at critical risk of bias.

Among the randomized trials, the most frequent concerns were related to the randomization process, missing outcome data, and the selection of the reported results. In several studies, insufficient information was provided regarding sequence generation and/or allocation concealment, leading to judgments of some concerns about bias arising from the randomization process. In addition, several trials were affected by post-randomization attrition or by complete-case analyses, thereby increasing the risk of bias from missing outcome data. These issues were particularly evident in studies with substantial dropout rates or small final analytical samples. Crossover trials required additional consideration of period effects, carryover effects, and washout adequacy; these design-specific issues contributed to less favorable judgments in crossover studies.

Of the randomized studies, Dukaew et al. [31] was judged to be the most methodologically robust, with low risk of bias across most domains and only minor residual concerns regarding selective reporting. By contrast, Coombes et al. [32], Lund et al. [33], Ooi et al. [28], Al-Nimer et al. [34], and Durkalec-Michalski et al. [35] were judged to be at high risk of bias, primarily due to attrition, limitations inherent to crossover design, or insufficiently documented randomization procedures. The remaining randomized trials were judged to have some concerns, reflecting moderate methodological limitations that were not sufficient to invalidate the findings entirely but warranted cautious interpretation.

All three non-randomized intervention studies were judged to be at critical risk of bias according to the ROBINS-I tool. The principal reasons for these judgments were the absence of a concurrent control group, substantial risk of confounding, open-label designs, and the use of simple pre–post comparisons that limited causal inference. Additional concerns included potential selection bias and selective outcome reporting. As a result, findings from the non-randomized studies were interpreted with considerable caution and were considered supportive rather than confirmatory within the overall evidence synthesis (Table 3).

Taken together, the risk-of-bias assessment indicates that the available evidence is limited by methodological weaknesses across several studies, particularly incomplete reporting of randomization procedures, attrition, and non-randomized study design. These limitations were taken into account when interpreting the direction, magnitude, and certainty of the observed effects.

**Table 1:** Risk-of-bias assessment of randomized controlled trials using RoB 2.

Study	Tool	D1	D2	D3	D4	D5	D6	Overall
Antonio et al. [36]	RoB 2	Some concerns	N/A	Low	Some concerns	Low	Some concerns	Some concerns
Coombes et al. [32]	RoB 2	Some concerns	N/A	Low	High	Low	Some concerns	High risk
Hofman et al. [37]	RoB 2	Some concerns	N/A	Low	Low	Low	Some concerns	Some concerns
Kerksick et al. [38]	RoB 2	Some concerns	N/A	Low	Low	Low	Some concerns	Some concerns
Lund et al. [33]	RoB 2 (crossover)	Some concerns	Some concerns	Low	High	Low	Some concerns	High risk
Duff et al. [39]	RoB 2	Some concerns	N/A	Low	Low	Low	Some concerns	Some concerns
Al-Nimer et al. [34]	RoB 2	High	N/A	Low	Low	Low	Some concerns	High risk
Ooi et al. [28]	RoB 2	Some concerns	N/A	Low	High	Low	Low	High risk
Dukaew et al. [31]	RoB 2	Low	N/A	Low	Low	Low	Some concerns	Some concerns
Durkalec-Michalski et al. [35]	RoB 2 (crossover)	Some concerns	High	Low	High	Low	Low	High risk

Risk of bias was assessed using the revised Cochrane risk-of-bias tool for randomized trials (RoB 2). For crossover trials, the RoB 2 adaptation for crossover designs was applied. The assessed domains were as follows: **D1**, bias arising from the randomization process; **D2**, bias due to period and carryover effects (crossover trials only); **D3**, bias due to deviations from intended interventions; **D4**, bias due to missing outcome data; **D5**, bias in measurement of the outcome; and **D6**, bias in selection of the reported result. Overall risk-of-bias judgments were assigned according to the RoB 2 guidance. Color coding was used to facilitate interpretation: **green**, low risk of bias; **yellow**, some concerns; **red**, high risk of bias; **grey**, not applicable (N/A).

**Table 2:** Risk-of-bias assessment of non-randomized intervention studies using ROBINS-I.

Study	Tool	D1	D2	D3	D4	D5	D6	Overall
Han et al. [29]	ROBINS-I	Critical	Low	Serious	Moderate	Moderate	Serious	Critical risk
Kim et al. [40]	ROBINS-I	Critical	Low	Serious	Moderate	Low	Serious	Critical risk
Mizrahi et al. [41]	ROBINS-I	Critical	Low	Serious	Moderate	Moderate	Serious	Critical risk

Risk of bias was assessed using the ROBINS-I tool (Risk of Bias in Non-randomized Studies of Interventions). The assessed domains were as follows: **D1**, bias due to confounding; **D2**, bias in the selection of participants into the study; **D3**, bias in the classification of interventions; **D4**, bias due to deviations from intended interventions; **D5**, bias due to missing data; **D6**, bias in the measurement of outcomes; and **D7**, bias in the selection of the reported result. Overall risk-of-bias judgments were assigned according to ROBINS-I guidance. Color coding was used to facilitate interpretation: **green**, low risk of bias; **yellow**, moderate risk of bias; **orange**, serious risk of bias; **red**, critical risk of bias.

**Table 3:** Study-level justification for the overall risk-of-bias judgments assigned to the included studies.

Study	Design	Overall Judgment	Rationale for Overall Risk-of-Bias Judgment
Antonio et al. [36]	Randomized, double-blind, placebo-controlled trial	Some concerns	The trial was described as randomized and double-blind, and the outcome assessment relied predominantly on objective measures, including DXA-derived body composition and standardized exercise testing. However, the report did not provide sufficient detail regarding the method used for sequence generation or the procedures for allocation concealment, precluding a low-risk judgment for bias arising from the randomization process. Furthermore, the publication did not clearly indicate whether the reported analyses were based on a prespecified statistical analysis plan or a predefined primary outcome, raising concerns about the selection of the reported results. No major concerns were identified regarding deviations from intended interventions, missing outcome data, or outcome measurement.
Coombes et al. [32]	Randomized, double-blind, placebo-controlled trial	High risk of bias	Although the study was reported as randomized, double-blind, and placebo-controlled, a substantial proportion of participants were excluded after randomization because of non-compliance with supplementation or training, and the final analysis was restricted to those who completed the protocol. This raises a high risk of bias due to missing outcome data, as the analytical sample may no longer have retained the balance conferred by randomization. In addition, insufficient detail was provided regarding the generation of the randomization sequence and the concealment of allocation, raising concerns in the randomization domain. Given the extent of post-randomization exclusion, the study was judged overall as being at high risk of bias.
Hofman et al. [37]	Randomized, double-blind, placebo-controlled trial	Some concerns	The study appeared adequately blinded and employed an active comparator with a comparable nutritional profile, with no major concerns identified regarding deviations from intended interventions, missing outcome data, or measurement of the outcome. Nevertheless, the report did not describe the methods used for sequence generation or allocation concealment in sufficient detail to support a low-risk judgment for the randomization process. In addition, it was unclear whether the reported analyses were prospectively specified. Accordingly, some concerns were raised about bias arising from the randomization process and the selection of the reported result.
Kerksick et al. [38]	Randomized, double-blind, placebo-controlled, multi-arm trial	Some concerns	This was a randomized, double-blind, multi-arm trial with standardized resistance training and objective assessments of body composition and performance. However, the publication did not explicitly describe the procedures used to conceal allocation, which resulted in some concerns regarding the randomization process. Moreover, multiple efficacy outcomes were reported across several physiological domains without a clearly stated prespecified hierarchy of primary and secondary outcomes. This raised concerns about selective reporting. As no major concerns were identified regarding deviations from intended interventions, missing data, or outcome measurement, the overall judgment was ‘some concerns’.
Lund et al. [33]	Randomized crossover trial	High risk of bias	In this randomized crossover trial, only 8 of the 12 enrolled participants were included in the final analysis, resulting in substantial attrition in a small clinical sample. This gave rise to a high risk of bias due to missing outcome data. In addition, the crossover design required consideration of period effects, washout adequacy, and possible carryover, and these issues could not be fully excluded on the basis of the information provided. Some concerns also remained regarding the randomization process, as the sequence generation and allocation procedures were insufficiently described. Taken together, these limitations justified an overall judgment of high risk of bias.
Duff et al. [39]	Randomized, double-blind trial	Some concerns	The study reported randomization and double-blinding, and the intervention was implemented alongside supervised resistance training, with repeated outcome assessment. No major concerns were identified for deviations from intended interventions, missing outcome data, or measurement of the outcome. However, the allocation concealment procedure was not described in sufficient detail, raising concerns about bias arising from the randomization process. In addition, the report did not fully clarify whether the reported outcomes and analyses were based on a prespecified analysis plan, raising concerns about the selection of the reported results.
Al-Nimer et al. [34]	Randomized, double-blind, placebo-controlled clinical study	High risk of bias	Although the trial was described as randomized and double-blind, the publication provided limited information regarding sequence generation and allocation concealment. Moreover, baseline imbalances were evident between the groups in several measured variables, raising concern that the randomization process may not have achieved adequate comparability or may have been insufficiently implemented or reported. The statistical presentation was also largely focused on within-group pre-post comparisons rather than robust between-group estimates, raising concerns about the reporting and interpretation of intervention effects. Because the randomization domain was judged to be at high risk, the overall study was judged to be at high risk of bias.
Ooi et al. [28]	Randomized, double-blind, placebo-controlled trial	High risk of bias	The study reported computerized randomization, blinding, and placebo control; however, 14 of the 66 randomized participants were excluded from the final analysis, and the published results were based solely on completers. Such attrition introduces a high risk of bias due to missing outcome data, particularly where the final estimates are not derived from a full intention-to-treat framework. Although no major concerns were identified regarding outcome measurement, the extent of attrition following randomization was sufficient to compromise confidence in the validity of the reported intervention effects.
Dukaew et al. [31]	Randomized, double-blind, placebo-controlled trial	Some concerns	This was the most methodologically rigorous randomized trial among the included studies. The authors reported computer-generated randomization, appropriate allocation concealment using sequentially numbered opaque sealed envelopes, double blinding, and prospectively defined endpoints. No major concerns were identified for bias arising from the randomization process, deviations from intended interventions, missing outcome data, or outcome measurement. However, because the full statistical analysis plan was not available in the published report, the possibility of selective reporting could not be excluded with complete certainty. Accordingly, the study was judged as presenting some concerns overall.
Durkalec-Michalski et al. [35]	Randomized, double-blind, placebo-controlled crossover study	High risk of bias	This crossover trial was strengthened by randomization, blinding, placebo control, and prospective trial registration. Nevertheless, substantial attrition occurred between enrollment and final analysis, resulting in a high risk of bias due to missing outcome data. In addition, crossover-specific methodological concerns, including period and sequence effects and the possibility of carryover despite the washout period, remained relevant and could materially affect the validity of the estimated treatment effect. As at least one key domain was judged at high risk, the overall study judgment was high risk of bias.
Han et al. [29]	Open-label, single-arm pilot intervention study	Critical risk of bias	This was an open-label, single-arm intervention study conducted without a concurrent control group. Under ROBINS-I, the absence of a valid comparator resulted in a critical risk of bias due to confounding, as any observed changes could not be distinguished from temporal trends, regression to the mean, co-interventions, or other uncontrolled influences. Additional concerns were identified regarding the selection of participants into the study and the selection of the reported results. In accordance with ROBINS-I guidance, the presence of critical risk in the confounding domain led to an overall judgment of critical risk of bias.
Kim et al. [40]	Single-arm before–and–after intervention study	Critical risk of bias	Although the article used the term ‘randomized’, the study was analytically equivalent to an uncontrolled single-arm before-and-after intervention, as no concurrent comparator group was included. Consequently, the study was judged at critical risk of bias due to confounding, because the observed metabolic changes could not be reliably attributed to the intervention rather than to time effects, behavioral changes, or other co-interventions. Additional concerns were present regarding participant selection and the selection of the reported result. In ROBINS-I terms, the absence of a valid counterfactual comparison is sufficient to justify an overall judgment of critical risk of bias.
Mizrahi et al. [41]	Open-label, single-arm phase I/II clinical trial	Critical risk of bias	This was a small, open-label, uncontrolled phase I/II clinical study involving only 10 participants. The absence of a concurrent control group resulted in a critical risk of bias due to confounding, as the reported improvements could not be disentangled from background temporal change or co-intervention effects. Further concerns arose regarding selection into the study and selection of the reported result, particularly because the interpretation emphasized favorable changes among subsets of participants. Consistent with ROBINS-I guidance, the study was therefore judged to be at critical risk of bias overall.

The table summarizes the primary methodological reason underlying the final judgment for each study.

### 3.2. Certainty of Evidence According to GRADE

The GRADE assessment showed that the certainty of evidence regarding the effects of bovine colostrum supplementation on body fat content and blood lipid profile was predominantly very low. The Summary of Findings according to the GRADE approach is presented in Table 4. Detailed GRADE evidence profiles and extracted numerical data used to support the certainty-of-evidence assessment are provided in Supplementary Materials (Tables S1–S4).

For body composition outcomes, including total fat mass, percentage body fat, and regional adiposity, most studies did not demonstrate a statistically significant advantage of bovine colostrum supplementation over placebo or protein-based comparators. Although individual studies reported favorable changes in lower-limb fat percentage or trends toward reduced adiposity, these effects were not consistently replicated across the included trials.

Similarly, the certainty of evidence for lipid profile outcomes, including TC, LDL-C, HDL-C, and TG, was rated as very low due to methodological heterogeneity, inconsistency of findings, imprecision related to small sample sizes, and indirectness resulting from differences in study populations and intervention protocols. Some studies conducted in participants with type 2 diabetes or older adults reported reductions in TC, LDL-C, or TG following bovine colostrum supplementation; however, these effects were not consistently confirmed in randomized controlled trials.

Overall, the currently available evidence is insufficient to support firm conclusions regarding the independent effect of bovine colostrum supplementation on reducing body fat content or improving blood lipid profiles in adults.

### 3.3. The Effect of Colostrum Bovinum (COL) Supplementation on Human Body Fat Content

In this subsection, the results of clinical studies evaluating the effects of bovine colostrum (COL) supplementation on body composition parameters, with particular emphasis on body fat content in adults, are presented (Table 5). Given the substantial heterogeneity of the available evidence, including differences in study populations, intervention duration, supplementation doses, the composition of comparator preparations, and the methods used for body composition assessment, the findings were analyzed primarily qualitatively. To ensure consistency throughout the review, groups receiving bovine colostrum were designated COL, whereas control or placebo groups were designated PLA, regardless of the type of comparator preparation used. In interpreting the findings, it was noted that the effects of COL on body fat content were generally assessed alongside other outcomes, including body weight, fat-free mass, muscle mass, exercise performance, and metabolic markers. Moreover, the observed effects may have depended not only on the supplementation itself but also on the participants’ level of physical activity, age, baseline status, and the presence of concomitant training or dietary interventions.

Antonio et al. [36] conducted an 8-week study to evaluate the effects of bovine colostrum (COL) supplementation on body composition, muscle strength, and muscular endurance in healthy, physically active adults. The study included 22 participants of both sexes, who were comparable in age, body weight, height, and body fat content at baseline, and were randomly assigned to two groups. The first group (*n* = 9) received 20 g/day of COL, whereas the second group (*n* = 13) received a placebo (PLA) containing 20 g of whey protein powder dissolved in liquids. During the intervention, participants performed aerobic and high-intensity resistance training at least three times per week. Dietary intake of macro- and micronutrients was assessed using three repeated 24-h dietary recalls. No significant differences were found between the COL and PLA groups with respect to weekly training frequency (aerobic and resistance exercise), the number of exercises, sets, repetitions, or the total estimated training volume (sets × repetitions) for resistance exercise. Likewise, no significant between-group differences were observed in dietary intake of macro- or micronutrients. However, supplementation had a significant effect (*p* < 0.05) on body weight, which increased in the PLA group (77.71 kg pre-intervention vs. 79.82 kg post-intervention), and a significant effect (*p* < 0.01) on lean body mass (LBM), which increased in the COL group (63.59 kg pre-intervention vs. 65.08 kg post-intervention). Nevertheless, body fat content in participants receiving COL (18.8% pre-intervention vs. 17.6% post-intervention) did not differ significantly from that observed in the PLA group (18.7% pre-intervention vs. 20.2% post-intervention). The authors attributed the lack of significant changes in body fat content to the absence of differences in energy and macronutrient intake between the groups. In their view, among already trained individuals, supplementation with 20 g/day of COL or PLA (whey protein), in the absence of systematic modifications to training or diet, may not constitute a sufficient stimulus to induce a significant reduction in adipose tissue over an 8-week period.

Coombes et al. [32] conducted an 8-week study in 42 Australian adult male competitive cyclists to examine the effects of dietary supplementation with bovine colostrum (COL) at doses of 20 g/day or 60 g/day, as well as 40 g/day of whey protein concentrate (WPC), on physical performance. Only participants without diagnosed lactose intolerance, cow’s milk protein intolerance, metabolic, vascular, respiratory, or other chronic diseases, and those not using dietary supplements were eligible for inclusion. However, 14 cyclists were excluded from the final analysis because of non-compliance with the supplementation or training protocol. Ultimately, the authors analyzed data from 28 cyclists, in whom two performance tests were performed before and after the supplementation period: VO_2_max assessment (Performance Measure One) on a cycle ergometer, followed by a 20-min recovery period, and Performance Measure Two, consisting of 2 h of cycling on a cycle ergometer at 65% of HRmax. Participants received their assigned supplements in 20-g sachets for 56 days. Supplementation was administered twice daily: a morning dose (20 g, dissolved in 85 mL of warm water and 40 mL of skim milk) and an evening dose (40 g, dissolved in 170 mL of warm water and 80 mL of skim milk). In addition, throughout the experimental period, the cyclists maintained both training logs and dietary records. Energy intake and macro- and micronutrient consumption during the intervention were assessed. Body composition was evaluated using the seven-site skinfold thickness method, including measurements at the biceps, triceps, subscapular, suprailiac, abdominal, quadriceps, and medial calf sites. Regarding body weight and body composition, no significant differences were observed between the cyclist groups based on the supplementation regimen. Across all groups, only a downward trend in the sum of skinfold thicknesses was noted between the pre- and post-supplementation assessments (PLA: 70 vs. 68 mm; COL 20 g: 69 vs. 64 mm; COL 60 g: 64 vs. 58 mm), which may indirectly reflect a reduction in adipose tissue. However, the authors did not report direct body fat content values. In the study by Coombes et al. [32] neither COL alone nor COL combined with WPC had a significant effect (*p* > 0.05) on VO_2_max improvement in cyclists (placebo: +3.4% vs. COL 20 g + WPC 40 g: +4.0%; COL 60 g: +3.9%). By contrast, cyclists completed the time-trial test significantly faster (*p* < 0.05) after the period of COL supplementation than before supplementation (performance improvement: placebo = 37 s; 20 g = 158 s; 60 g = 134 s). The authors concluded that COL supplementation, possibly through improved nutrient absorption in the small intestine, significantly enhanced time-trial performance in cyclists following 2 h of cycling at 65% VO_2_max, suggesting improved muscle cell recovery or greater metabolic efficiency. However, unlike previous reports [42], authors did not confirm that COL supplementation increased plasma IGF-1 concentrations or enhanced post-recovery anabolic processes, thereby contributing to greater overall training adaptation.

Hofman et al. [37] performed an 8-week study in 35 elite field hockey players, including members of the Dutch national team (17 women and 18 men), to examine the effects of dietary supplementation with bovine colostrum (COL; 60 g/day) or whey protein placebo (PLA; 60 g/day) on body composition and exercise performance. Body composition was assessed by measuring skinfold thickness at four sites (biceps, triceps, subscapular, and suprailiac), while physical performance was evaluated using the 5 × 10 m sprint, vertical jump, shuttle run, and suicide run tests. The COL and PLA supplements were comparable in terms of energy and nutritional value per 100 g (respectively: 376 vs. 379 kcal, 75 vs. 77 g protein, 11 vs. 11 g carbohydrates, and 3.5 vs. 3.0 g fat). Ultimately, the final analysis included 28 athletes (14 men and 14 women), with 7 participants excluded; only one was excluded for whey protein intolerance. It was found that over the 8-week supplementation period, both body weight and lean body mass (LBM) increased significantly in both groups. In the COL group, body weight increased significantly (*p* < 0.01) by 1.1 ± 0.3 kg, and LBM increased by 1.3 ± 0.3 kg. Similarly, in the PLA group, body weight increased significantly (*p* < 0.01) by 1.0 ± 0.3 kg, and LBM by 1.2 ± 0.3 kg. However, despite these significant increases in body weight and LBM, supplementation did not result in statistically significant changes in fat mass (a decrease of −0.2 ± 0.4 kg in both groups) or in the sum of skinfold thicknesses (−3.4 ± 1.6 mm vs. −3.9 ± 1.8 mm, respectively). No significant between-group differences were observed in these parameters. Regarding performance outcomes, a significant (*p* < 0.05) improvement in sprint time was observed in the COL group compared with the PLA group (0.64 ± 0.09 s vs. 0.33 ± 0.09 s, respectively). In contrast, no significant improvement was observed in either group or between groups in the shuttle run (endurance), suicide run (anaerobic endurance), or vertical jump tests. The authors suggested that the differential effect of COL supplementation on exercise tasks relying on the ATP–CP system (i.e., sprint and vertical jump) and those dependent on the aerobic/lactic acid energy system (i.e., shuttle run and suicide run) may have been related to the type of training undertaken, as the ATP–CP system had been trained to a greater extent than the aerobic energy system. In the authors’ view, the reduction in sprint time following COL supplementation may indicate a faster rate of dephosphorylation of creatine phosphate (CP) and ATP.

Kerksick et al. [38] examined the effects of bovine colostrum (COL), as compared with an isocaloric, isonitrogenous whey–casein blend supplemented with creatine (CR), on body composition, muscular strength and endurance, and anaerobic performance during a 12-week resistance training intervention in 49 healthy adults (36 men and 13 women) aged 18–45 years, all of whom had engaged in resistance training for more than one year. Participants were not using any dietary supplements or anabolic steroids. Prior to the intervention, their health status, body composition, and dietary habits were assessed, and they were assigned to four experimental groups matched for age and fat-free mass (FFM). Participants supplemented their habitual diet with one of the following regimens: protein control (PRO), PRO/COL, PRO/CR, or COL/CR. All supplements were isocaloric and isonitrogenous, providing 60 g/day of either casein/whey protein (PRO) or COL as the primary protein source. Body weight was measured at weeks 0, 8, and 12 of supplementation, and body composition was assessed using dual-energy X-ray absorptiometry (DXA). In addition, one-repetition maximum (1RM) and 80% 1RM bench press and leg press tests, as well as 30-s anaerobic sprint performance tests, were performed. The study demonstrated that resistance training increased 1RM strength, muscular endurance, and anaerobic sprint performance to a similar extent across all groups. Mild adverse effects of supplementation (i.e., bloating, cramping, and diarrhea) were reported by fewer than 10 participants, and these did not affect adherence to either the training program or the supplementation protocol. No significant differences were observed in the energy or nutritional value of the participants’ diets. However, significant main and interaction effects (*p* < 0.05) were found for body weight, total DXA mass, and FFM. No significant changes were observed in total body water (TBW), fat mass (FM), percentage body fat, or bone content. Participants in the PRO/COL, PRO/CR, and COL/CR groups showed greater increases (*p* = 0.02) in body weight and total DXA mass than those in the PRO group. Moreover, individuals receiving PRO/CR and COL/CR exhibited greater increases (*p* < 0.05) in DXA-derived FFM during training compared with the PRO group (*p* = 0.04). The authors attributed this finding to the differential physiological effects of the protein fractions: whey protein promotes rapid amino acid release, thereby stimulating muscle protein synthesis, whereas casein digestion in the gastrointestinal tract leads to slower amino acid release, which may help reduce protein breakdown. According to the authors, the combination of whey and casein may therefore have contributed to the increases in FFM observed in these groups [43]. In summary, the authors concluded that, over 12 weeks of resistance training, creatine was a key contributor to gains in muscle mass and strength, whereas the combination of bovine colostrum and protein did not confer any additional benefit compared with the standard whey–casein mixture.

Han et al. [3] conducted a 12-week pilot study in 13 healthy individuals aged 18–45 years, both sexes, with a BMI of 19–30 kg/m^2^, to assess the safety and efficacy of the multicomponent dietary supplement RiteStart^®^, formulated in sex-specific versions for women and men. In addition to bovine colostrum (COL), the supplement contained, among other ingredients, vitamins, minerals, oligomeric proanthocyanidins derived from pine bark, grape seed extracts, lutein, coenzyme Q10 (CoQ10), α-lipoic acid, green tea, essential fatty acids from fish and plant oils, egg yolk extract, maitake mushrooms, shiitake mushrooms, cordyceps, inositol hexaphosphate, and olive leaf extract. During four study visits, the effects of supplement intake were evaluated with respect to anthropometric parameters (body weight and height), body composition (the method and equipment used were not specified), as well as hematological, biochemical, and enzymatic blood parameters, including ALT, AST, GGT, creatine kinase, glucose, and CRP. In addition, the study assessed concentrations of vitamins (folate, B12, and D), minerals (iron, magnesium, potassium, and sodium), hormones (testosterone, free testosterone, DHEAS, cortisol, and SHBG), the lipid profile (TC, HDL-C, LDL-C, and TG), salivary parameters (sIgA), and general health indicators such as blood pressure and heart rate. The study demonstrated a significant increase in glucose concentration following supplementation (77.69 ± 7.11 vs. 94.08 ± 6.92 mg/dL), which the authors attributed to a moderate increase in body weight, percentage body fat, and fat mass, although these changes were not considered statistically significant at the threshold of *p* ≤ 0.01. However, given the differences reported by the authors between baseline (week 0) and the end of the study (week 12) for fat mass (FM) (43.3 ± 24.6 vs. 45.3 ± 23.2 lb; *p* = 0.016), total body fat percentage (TFAT) (24.0 ± 12.0 vs. 25.5 ± 10.3%; *p* = 0.016), and total fat mass (TFM) (22.2 ± 13.8 vs. 23.8 ± 12.5 lb; *p* = 0.016), these changes could reasonably be regarded as statistically significant at the *p* < 0.05 level. The remaining findings from this study are discussed in Section 3.2.

Durkalec-Michalski et al. [35] carried out a 12-week study incorporating both a supplementation phase and a washout period in a final cohort of 28 healthy men aged 30–40 years who were engaged in moderate triathlon and swimming training. Initially, 58 participants were enrolled; however, 30 were excluded because of injury or illness. The study aimed to assess the effects of bovine colostrum (COL; 25 g/day; *n* = 13) versus placebo (PLA; 25 g/day; *n* = 15) on aerobic capacity (graded exercise test performed on a cycle ergometer/treadmill), body composition (assessed by bioelectrical impedance analysis, BIA), and blood lactate concentration measured both at rest (REST) and post-exercise (POST-IRT). A 25 g serving of either COL or PLA provided approximately 89 kcal, 4.5 g of carbohydrates, and ≤17.5 g of protein. The authors found no significant effect of supplementation on body weight in either the COL group (82.2 ± 9.4 vs. 82.5 ± 9.4 kg, pre- vs. post-intervention) or the PLA group (82.0 ± 8.8 vs. 82.7 ± 9.5 kg) among these trained men. Likewise, supplementation did not induce significant changes in total body water (TBW), fat-free mass (FFM), or fat mass (FM) in either group. In the COL group, the respective pre- and post-intervention values were 57.3 ± 4.7 vs. 58.8 ± 4.9% for TBW, 82.1 ± 5.8 vs. 82.5 ± 6.0% for FFM, and 17.9 ± 5.7 vs. 17.5 ± 6.0% for FM. Corresponding values in the PLA group were 58.6 ± 4.9 vs. 58.7 ± 5.4%, 83.2 ± 5.3 vs. 82.2 ± 6.8%, and 16.7 ± 5.2 vs. 18.5 ± 7.1%, respectively. By contrast, the authors reported that COL supplementation significantly (*p* < 0.05) increased time to ventilatory threshold (TVT) and improved VO_2_ at ventilatory threshold (VO_2_VT) compared with PLA. However, COL supplementation did not significantly affect time to exhaustion (TEXH) during the incremental exercise test (IRT) or blood lactate concentrations. According to the authors, the effectiveness of COL supplementation in improving aerobic fitness, exercise capacity, and time to exhaustion may depend on its individualized integration into training cycles and on careful consideration of treatment sequence in long-term crossover protocols.

Studies on bovine colostrum (COL) supplementation have also been conducted in older adults. Duff et al. [39] carried out an 8-week study in 40 older participants (15 men and 25 women, mean age approximately 59 years) to evaluate the effects of COL supplementation, compared with whey protein (PLA), on muscle strength, body composition, inflammatory markers, and bone turnover during a standardized resistance training program (three sessions per week, 12 exercises per session). Participants were allocated to two groups: COL (12 women, 7 men) and PLA (13 women, 8 men). They consumed 60 g/day of either COL or whey protein (approximately 38 g of pure protein), divided into three 20 g doses: one taken within 30 min before exercise, another within 30 min after exercise, and the third at the participant’s discretion. The COL and PLA supplements were comparable in energy and nutritional composition per 100 g (respectively: 429 vs. 441 kcal; 62.4% vs. 64.6% protein; 13.9% vs. 14.7% fat; 13.5% vs. 12.5% carbohydrates). Assessments performed before and after supplementation included body composition measured by DXA, muscle thickness of the biceps and quadriceps assessed by ultrasonography, strength testing using 1-RM bench press and leg press, cognitive performance evaluated with the Telephone Interview of Cognitive Status (TICS), bone resorption based on urinary *N*-telopeptide (Ntx) excretion, and serum concentrations of IGF-1 and CRP. However, the final analysis included 37 participants, as several individuals withdrew for personal reasons or because of severe gastroesophageal reflux, flatulence, or bloating in both groups. Significant changes were observed after the supplementation period in both groups. With respect to muscle strength, both groups showed significant improvements in the bench press; however, in the leg press, the COL group achieved a significantly greater (*p* < 0.05) increase in strength (24 ± 29 kg) than the PLA group (8 ± 16 kg). Regarding body composition, both groups demonstrated significant (*p* < 0.05) increases in lean tissue mass (+0.7 kg in the COL group; +0.5 kg in the PLA group), bone mineral content (+0.03 kg vs. +0.01 kg), biceps muscle thickness (+0.27 cm in both groups), and quadriceps muscle thickness (+0.22 cm in the COL group vs. +0.21 cm in the PLA group). At the same time, percentage body fat decreased by 0.4% in both groups. Protein intake also declined significantly (*p* < 0.05) during supplementation in both groups. No supplementation-related changes in serum IGF-1 or CRP concentrations were observed in either group. However, between-group analysis showed a significantly greater (*p* < 0.05) reduction in the urinary bone resorption marker (Ntx cross-linked *N*-telopeptides of type I collagen) in the COL group than in the PLA group. Cognitive test scores (TICS) improved significantly over time in both groups, with no between-group differences, which the authors attributed either to the exercise intervention itself or to a practice effect related to repeated testing. Sex-based analyses revealed that, compared with women, men had significantly (*p* < 0.05) greater leg press and bench press strength, higher serum IGF-1 concentrations, greater bone mineral content, higher lean body mass, greater elbow flexor and knee extensor muscle thickness, and higher energy, protein, and carbohydrate intake. Men also performed better on cognitive tests than women, despite having lower body fat percentages. In summary, the authors concluded that COL supplementation, when combined with a resistance training program, improved leg press performance and reduced bone resorption compared with PLA (whey protein). However, COL did not demonstrate superiority over PLA for bench press performance, muscle mass, bone mass, IGF-1, CRP, or changes in body weight, as these parameters improved similarly in both groups. The observed improvement in cognitive function was most likely attributable to the training intervention itself rather than to the type of supplement consumed. According to the authors, the effect of COL on lower-body strength (an increase of 21%) and bone health may be of practical relevance in older adults, in whom declines in leg strength and bone mass are major risk factors for falls, fractures, and frailty. They further emphasized that the greater increase in leg strength is clinically meaningful, given that functional decline in this domain is strongly associated with physical limitations in older individuals.

A clinical study involving 80 older adults (55–70 years) of both sexes was also conducted by Dukaew et al. [31], to evaluate the effects of a colostrum-containing multi-nutrient supplement product (COL), compared with placebo (PLA), on various parameters associated with the aging process. The study was based on the concept of “healthy aging,” which has attracted increasing attention as an approach to improving health, enhancing well-being, and preserving functional independence in later life. Participants enrolled in the study and assigned to two groups (COL, *n* = 40; PLA, *n* = 40) had a BMI of 18.5–22.9 kg/m^2^, were in good physical health, and exhibited either normal cognitive function or only mild cognitive impairment. During the 12-week intervention, participants in the COL group consumed two sachets daily (100 g in total), dissolved in 180 cm^3^ of water and divided into morning and evening doses. One sachet (50 g) of the COL supplement contained 5 g of bovine colostrum, 14 g of whey protein isolate, 4 g of soy protein isolate, 0.1 g of rice protein, 0.5 g of choline, and a vitamin–mineral premix. The PLA supplement, in contrast, contained the same total amount of protein and excipients but lacked the active ingredients, namely colostrum, calcium, vitamins, and choline. Measurements performed at baseline (week 0) and again at weeks 4 and 12 included parameters of immune function and inflammation (fecal bovine IgG, salivary sIgA, serum human IgG, complete blood count, T- and B-lymphocyte subpopulations, IL-6, TNF-α, IL-10, and CRP), blood biochemical markers (IGF-1), body composition, bone density and bone-related parameters (BMC, BMD, P1NP, osteocalcin, and β-CrossLaps), and cognitive function assessed using the Thai Mental State Examination (TMSE) and the Montreal Cognitive Assessment, Thai version (MoCA-Thai). Adverse events were also monitored. At baseline, participants in both groups had comparable values for the assessed parameters. After the 12-week intervention, participants receiving COL showed a significant (*p* < 0.0001) increase in fecal bovine IgG concentration (+2.75 μg/cm^3^), confirming that active bovine immunoglobulins passed through the gastrointestinal tract and may have enhanced passive mucosal immunity in the gut. However, this did not translate into a significant increase in circulating human IgG antibodies, as the between-group difference in serum human IgG was 0.50 μg/cm^3^ and did not reach statistical significance (*p* = 0.0964). Participants in the COL group also had a significantly (*p* = 0.0085) lower percentage of eosinophils (−1.03%) than those in the PLA group. In addition, the COL group exhibited a significant (*p* < 0.05) increase in serum IGF-1 concentration of 10.30 ng/cm^3^ relative to PLA, which may potentially mitigate the age-related decline in growth hormone activity and reduce the risk of frailty and disability in older adults. With respect to body composition, COL supplementation, compared with PLA, was associated only with a significantly (*p* < 0.05) smaller increase in the percentage of fat tissue in the legs (0.48 ± 1.29% vs. 1.12 ± 1.27%, respectively). The lack of changes in the remaining body composition parameters, including BMI, total mass, total body fat, total lean mass, arm and leg lean mass, and RSMI, was explained by the authors as likely reflecting the fact that COL supplementation alone—despite providing protein and increasing IGF-1—is probably an insufficient stimulus to induce meaningful increases in muscle mass or reductions in fat mass in the absence of concurrent resistance training. Moreover, the participants were in good baseline health, without overt deficiencies or functional impairments, which limited the likelihood of observing marked improvement over a short period. The 12-week intervention may also have been too brief to detect tissue remodeling processes that require longer-term adaptation. Thus, the increase in IGF-1 may primarily have supported repair and immune-related processes, rather than directly stimulating muscle accretion in the absence of an additional mechanical stimulus such as physical exercise. The study did not demonstrate a significant effect of COL supplementation on BMD, bone turnover markers (P1NP, osteocalcin, β-CrossLaps), immune and inflammatory markers (human IgG, IL-10, WBC count, or leukocyte subtypes including neutrophils, basophils, monocytes, and lymphocytes), the incidence of upper respiratory tract infections, or the frequency of adverse events, indicating that both preparations were well tolerated. Likewise, no significant between-group differences in cognitive function were observed, as assessed by either the TMSE or the MoCA. In summary, the clinically relevant benefits observed in these older participants following 12 weeks of safe supplementation with a multicomponent preparation containing bovine colostrum, compared with PLA, included increased fecal IgG and serum IGF-1 concentrations, together with a modest but significant reduction in eosinophil percentage and lower-limb fat accumulation. These effects may be relevant to promoting healthy aging in older adults.

The studies discussed above included healthy individuals or healthy physically active participants (both younger adults and older adults). In contrast, Lund et al. [33] conducted a study in 12 adult patients (5 women and 7 men, mean age 55.7 ± 10.7 years) with short bowel syndrome (SBS), including 9 patients with intestinal failure and 3 with relative intestinal insufficiency. The study aimed to evaluate the effects of 4-week dietary supplementation with bovine colostrum (COL; 250 cm^3^/day) or an isoenergetic and isoprotein placebo (PLA; 250 cm^3^/day), consumed in place of water, on intestinal adaptation and function, with the two intervention periods separated by a 4-week washout phase during which participants followed only their habitual diet. In patients with this clinical condition, effective strategies to improve enteral nutrient delivery and thereby stimulate adaptation of the remnant bowel are still being sought. Accordingly, the authors investigated whether COL would improve intestinal adaptation and function in adults with SBS, as compared with a standard milk protein–based placebo (PLA). In the study, COL and PLA had broadly similar energy and nutrient values per 100 cm^3^. Their respective contents of energy, protein, carbohydrates, and fat were 534 kJ, 10.6 g, 3.6 g, and 5.5 g for COL, and 455 kJ, 1.5 g, 4.8 g, and 3.2 g for PLA. Four participants were excluded from the final analysis because of nausea and vomiting after COL consumption, personal reasons, or dental problems that prevented adherence to the prescribed diet. The study assessed fluid, electrolyte, and macronutrient balance during 72-h hospital stays with weighed food records, and also included chemical analyses of stool and urine, body composition assessment by DXA, functional testing (handgrip strength), spirometry, and measurement of maximal inspiratory and expiratory pressures. In addition, blood chemistry parameters and glucagon-like peptide-2 (GLP-2) were measured, although not all results were presented in the article. Both COL and PLA significantly increased absolute protein absorption (0.96 ± 0.42 MJ/day vs. 1.03 ± 0.44 MJ/day, respectively) and energy absorption (1.46 ± 1.02 MJ/day vs. 1.76 ± 1.46 MJ/day, respectively) relative to baseline values. With regard to body composition, no significant changes were observed in the COL group in either body weight (0.0 ± 1.6 kg) or lean body mass (LBM) (−0.35 ± 1.9 kg). In contrast, in the PLA group, body weight increased significantly by 0.9 ± 0.3 kg, and LBM by 1.1 ± 1.3 kg. However, neither supplement had a significant effect on total fat mass (COL: 16.83 ± 9.6 kg vs. PLA: 16.81 ± 9.3 kg). At least three factors may explain the absence of meaningful changes in body composition. First, the 4-week supplementation period may have been too short to induce measurable changes in patients with SBS. Second, because both supplements were isoenergetic and isoprotein, their effects on whole-body energy balance were likely similar. Third, the increased energy and protein absorption associated with supplementation may have exacerbated diarrhea in these patients through an osmotic effect, thereby limiting the actual utilization of absorbed nutrients for tissue accretion. In terms of physical performance and functional outcomes, dominant handgrip strength improved significantly (*p* < 0.05) after both supplements (COL: 3.1 ± 3.3 kg; PLA: 2.6 ± 2.8 kg). No significant changes were observed in pulmonary function tests or basal metabolic rate (BMR). Both supplements also caused a significant increase in wet stool output (COL: 231 ± 248 g/day; PLA: 319 ± 299 g/day), which the authors attributed to the increased osmotic and protein load. When administered with breakfast, neither supplement increased plasma GLP-2 concentrations. In summary, the authors concluded that, despite the richness of bioactive components in bovine colostrum, it was not superior to the standard milk-based control preparation (PLA) in improving intestinal absorption, body composition, or functional test performance in adult patients with short bowel syndrome. However, given the small study group, the authors’ results should be considered ambiguous due to low statistical power.

### 3.4. The Effects of Colostrum Bovinum (COL) Supplementation on Human Blood Lipid Profile

In this subsection, the findings of clinical studies evaluating the effects of bovine colostrum (COL) supplementation on the blood lipid profile in humans are presented, with particular emphasis on triacylglycerol (TG), total cholesterol (TC), and its LDL-C and HDL-C fractions (Table 6). Given the substantial heterogeneity of the available studies, including differences in participants’ health status, the composition of the preparations administered, the duration of the intervention, and the range of metabolic parameters assessed, the findings were interpreted primarily qualitatively. In most of the studies analyzed, the lipid profile was one of several endpoints evaluated in parallel with parameters related to glycemic control, inflammatory status, and overall metabolic status.

Kim et al. [40] conducted a 4-week study in 24 patients aged 35–65 years, both sexes, with type 2 diabetes mellitus to evaluate the effects of bovine colostrum (COL) supplementation on glycemic control and lipid profile. Participants consumed 5 g/day of powdered COL, administered in the morning on an empty stomach and again in the evening. The chemical composition of the supplement was analyzed, and the COL used in the study was obtained from cows within 6 h postpartum. At weekly intervals, blood samples were collected and analyzed using reflectance photometry and electrochemistry to determine concentrations of glucose (fasting, 2 h postprandial, and 8 h postprandial), triacylglycerols (TG), total cholesterol (TC), and β-hydroxybutyric acid. A reduction in blood glucose concentration at both 2 and 8 h after a meal was observed in participants of both sexes throughout the intervention. However, only the rate of decline in glucose concentration 2 h postprandially differed significantly between women and men, with values of 14.25 ± 2.66% and 10.96 ± 1.82%, respectively. After 4 weeks of COL supplementation, both TC and TG concentrations decreased significantly in both men and women (8.27 ± 1.43% vs. 7.62 ± 0.75%, and 11.96 ± 2.58% vs. 21.46 ± 3.53%, respectively). β-Hydroxybutyric acid concentrations were also lower in both sexes; however, this difference did not reach statistical significance. Authors concluded that COL supplementation exerts beneficial health effects in patients with type 2 diabetes mellitus, with effects appearing more pronounced in women. In their view, COL may serve as an effective supplement that favorably influences carbohydrate and lipid metabolism, thereby directly reducing the risk of cardiovascular complications in diabetic patients and the risk of developing ketoacidosis. According to the authors, the observed effects may be attributable to the actions of growth factors present in COL (e.g., IGF-1) and immunoglobulins.

Misrahi et al. [41] carried out a 30-day study in 10 adults of both sexes, aged 18–60 years, with type 2 diabetes mellitus and non-alcoholic steatohepatitis (NASH) confirmed by liver biopsy, to evaluate the safety and efficacy of oral therapy with Imm124-E, a biotechnological preparation containing hyperimmune bovine colostrum enriched with antibodies directed against *Escherichia coli* lipopolysaccharide (LPS). This phase I/II study was designed as a preliminary assessment of whether the preparation could influence insulin resistance, lipid profile, inflammatory and metabolic markers, and the number of regulatory T cells (Tregs). The colostrum used in Imm124-E was obtained from cows immunized against LPS, an endotoxin produced by intestinal bacteria. In patients with NASH, LPS may translocate into the circulation and trigger systemic and hepatic inflammation; therefore, Imm124-E was intended to neutralize these endotoxins by binding them directly in the intestine, preventing their passage into the portal vein and subsequently into the liver. Before and after treatment with Imm124-E, the following parameters were assessed: glycemic indices (HbA1c, fasting glucose concentration, oral glucose tolerance test [OGTT], and HOMA-IR); lipid profile (TC and LDL-C); inflammatory and metabolic markers (CRP, IL-6, GLP-1, and adiponectin); immunological parameters (regulatory T-cell counts determined by flow cytometry); additional laboratory markers (complete blood count, ALT, AST, γ-GT, alkaline phosphatase [AP], and bilirubin); and adverse events were monitored throughout the study. Following the treatment period, administration of Imm124-E significantly reduced HbA1c by approximately 14.8% on average, from 7.49% to 6.38% across all participants (*p* < 0.03). About other indices of glucose metabolism, only non-significant downward trends were observed, including reductions in fasting glucose concentration (6.3 vs. 5.8 mmol/L) and improvements in serum insulin concentration (310 vs. 538.4 pmol/L) in five patients, OGTT results (2492 vs. 2252) in five patients, and HOMA scores (6.7 vs. 4.8) in six patients. Increased post-OGTT serum GLP-1 concentrations were also noted in five treated patients (6.31 vs. 6.78 × 10^4^ pM), although these changes did not reach statistical significance. In addition, a significant (*p* = 0.002) increase in certain subtypes of regulatory T lymphocytes, specifically CD4^+^CD25^+^HLA-DR^+^ Tregs, was observed in seven patients, rising from 2.3% to 3.8%, which may reflect an anti-inflammatory immunomodulatory effect. By contrast, no significant changes were found in serum IL-6 concentrations (4.6 vs. 5.5 pg/mL in responders) or adiponectin concentrations, which, although increased in eight patients (6181 vs. 7069 ng/mL), did not change significantly. A reduction, albeit not statistically significant, was also observed in the activity of liver enzymes, including ALT, AST, AP, and γ-GT, after the treatment period (57.4 vs. 48.6 μ/L; 51.2 vs. 44.6 μ/L; 83.1 vs. 73.9 μ/L; 88.2 vs. 73.2 μ/L, respectively). Likewise, improvement in the lipid profile observed in five patients did not reach statistical significance, either for total cholesterol (5.8 vs. 4.5 μmol/L) or for LDL-C (5.8 vs. 4.5 μmol/L). No results were reported for triacylglycerols. Authors concluded that Imm124-E is safe, as no treatment-related adverse events were reported, and exerts immunomodulatory effects with the potential to improve glycemic control. This conclusion was based on the observed improvement in insulin resistance and hyperlipidemia, together with changes in cytokine levels and several Treg subpopulations.

Han et al. [29] conducted a 12-week pilot study involving 13 healthy individuals aged 18–45 years, both sexes, to evaluate the safety and efficacy of the multicomponent dietary supplement RiteStart^®^, which contained, among other ingredients, bovine colostrum (COL). In addition to the previously described blood parameters assessed in the participants (Section 3.1), the study also measured total cholesterol (TC), HDL-C, LDL-C, and triacylglycerols (TG) as part of the lipid profile. No significant effect of supplementation was observed between week 0 and week 12 on serum concentrations of TG (160.85 ± 78.33 vs. 177.62 ± 110.40 mg/dL; *p* = 0.524), TC (192.62 ± 29.11 vs. 194.08 ± 29.51 mg/dL; *p* = 0.816), HDL-C (43.85 ± 8.32 vs. 45.85 ± 9.75 mg/dL; *p* = 0.134), or LDL-C (121.31 ± 23.54 vs. 113.62 ± 19.47 mg/dL; *p* = 0.104). By contrast, salivary IgA concentrations increased significantly at weeks 4, 8, and 12 of the study (177.57 ± 74.81 vs. 249.85 ± 95.63 vs. 271.65 ± 133.52 vs. 279.88 ± 128.19 ng/mL, respectively). Concerning hematological parameters, supplementation was associated with a significant (*p* < 0.01) decrease in RBC count (5.34 ± 0.50 vs. 5.18 ± 0.48 × 10^6^/μL), mean corpuscular volume (MCV) (87.32 ± 4.10 vs. 89.98 ± 4.50 fL), and red cell distribution width (RDW) (13.08 ± 0.74% vs. 13.68 ± 1.00%). Supplementation also significantly (*p* ≤ 0.01) increased serum folic acid concentration by 48.3% (15.88 ± 3.40 vs. 23.56 ± 5.75 ng/mL). In contrast, the observed increases in vitamin B12 concentration (+21.3%) and serum 25-hydroxyvitamin D concentration (+9.1%) were not statistically significant. Sex hormone-binding globulin (SHBG) increased significantly (27.92 ± 6.17 vs. 36.77 ± 11.79 nmol/L), although values remained within the normal range for both sexes. Serum albumin concentration decreased significantly after supplementation (4.77 ± 0.26 vs. 4.57 ± 0.28 g/dL), but also remained within the reference range. No significant changes were observed in the activity of liver enzymes (ALT, AST, GGT), serum hsCRP concentration, or the levels of the assessed minerals (iron, potassium, sodium). According to the authors of this pilot study, the absence of any adverse effects of RiteStart^®^ on liver enzyme activity or on most hematological parameters supports its safety and good tolerability. They concluded that the supplement may effectively increase the levels of important micronutrients, particularly folate, and support immune function by enhancing salivary IgA secretion.

Al-Nimer et al. [34] conducted an 8-week study in 50 adult men from Iraq, aged 18–25 years, who were engaged in resistance training, to evaluate the effects of bovine colostrum (COL) supplementation on glucose metabolism under conditions of physical stress. Participants were assigned to two groups: a PLA group (*n* = 24), which received 500 mg/day of placebo (the composition of the placebo was not specified), and a COL group (*n* = 26), which received 500 g in a single oral nutraceutical pill. The effects of supplementation in these trained men, who performed 2-h resistance training sessions three times per week, were assessed based on anthropometric characteristics (body weight, height, waist circumference [WC], and BMI), glycemic and hematological parameters (fasting blood glucose, HbA1c%, TG, hemoglobin [Hb], and mean corpuscular volume [MCV]), hemodynamic parameters (blood pressure, heart rate, and rate pressure product [RPP]), and calculated hematological and glycemic indices (SHR/SGR, stress-FBG, and TyGI). After 8 weeks of supplementation combined with resistance exercise, no significant between-group differences were observed in BMI or WC; however, a significant (*p* < 0.001) reduction in MCV was noted. In addition, the PLA group showed a significant (*p* < 0.05) increase in RPP (+13.0%), whereas the COL group exhibited an increase in Hb concentration (+2.8%). In the PLA group, no significant changes were found in fasting blood glucose (+1%), HbA1c (+4.3%), or TyGI (+1.2%). Similarly, in the COL group, no significant difference in fasting blood glucose was observed (−1.1%), whereas HbA1c increased significantly by 10.6% (*p* < 0.001). Compared with the COL group, the PLA group had significantly (*p* < 0.001) lower values of the calculated fasting blood glucose index representing stress hyperglycemia (92.7 ± 13.9 vs. 102.1 ± 11.5), lower SHR (0.956 ± 0.164 vs. 1.18 ± 0.13), and significantly (*p* < 0.05) lower TyGI values (8.6 ± 0.47 vs. 9.1 ± 0.442). Results for triacylglycerols alone were not reported. Supplementation of colostrum during an 8-week resistance training program appeared to increase the stress hyperglycemia ratio (SHR) and elevate stress-related glucose concentration, while not adversely affecting metabolic indices (BMI, WC, TyGI) and stabilizing the hemodynamic response, as evidenced by the absence of a significant rise in RPP, in contrast to the PLA group. According to the authors, COL may provide an additional source of glucose required by skeletal muscle during intense exercise without altering the glucose–lipid profile in healthy young men. However, it should be noted that, already at baseline, participants in the PLA group had significantly (*p* < 0.05) lower WC, RPP, and TyGI values than those in the COL group (86.2 ± 7.6 vs. 91.6 ± 9.6 cm; 95.8 ± 19.9 vs. 119.8 ± 36.3; 8.4 ± 0.42 vs. 8.9 ± 0.38, respectively), which warrants caution in the interpretation of these findings.

Ooi et al. [28] also conducted a 12-week study in a final cohort of 52 older adults aged 50–69 years (23 men and 29 women) to evaluate the effects of consuming pasteurized milk powder enriched with bovine colostrum (COL), compared with pasteurized milk powder placebo (PLA), on anthropometric, hematological, biochemical, physical, cognitive, and quality-of-life parameters. Participants consumed the supplement twice daily, with each serving containing 15 g of powder and 150 mg of IgG. The COL group included 26 participants, and the PLA group also included 26 participants. The assessed anthropometric parameters comprised body weight, height, hip circumference, waist circumference, and BMI. In addition, the study evaluated blood pressure, hematological indices, biochemical blood parameters (lipid profile and glucose), physical performance using the Timed Up-and-Go (TUG) test and handgrip dynamometry, cognitive function (global cognition, psychomotor speed, verbal memory, assessed with the MMSE, RAVLT, Digit Span, and Digit Symbol tests), and quality of life using the WHOQOL-BREF questionnaire. At baseline, the two groups did not differ significantly in any of these parameters. With respect to anthropometric outcomes, both the COL and PLA groups exhibited a significant (*p* < 0.05) reduction in BMI from pre- to post-supplementation (26.55 ± 5.16 vs. 25.96 ± 4.79 kg/m^2^ and 27.84 vs. 27.06 ± 4.05 kg/m^2^, respectively). A reduction in hip circumference was also observed in both groups (100.49 ± 8.62 vs. 98.90 ± 8.26 cm and 104.89 ± 8.58 vs. 102.35 ± 9.57 cm, respectively), whereas a reduction in waist circumference was noted only in the PLA group (92.81 ± 8.60 vs. 88.06 ± 8.38 cm). Regarding the lipid profile, participants in the COL group showed a significant (*p* < 0.05) decrease in total cholesterol (TC) (5.88 ± 1.56 vs. 5.38 ± 1.19 mmol/L) and LDL-C (3.68 ± 1.47 vs. 3.28 ± 1.14 mmol/L) following supplementation, whereas those in the PLA group exhibited a reduction in HDL-C (1.42 ± 0.32 vs. 1.32 ± 0.27 mmol/L). No significant changes in TG or blood glucose concentrations were observed in either group. In terms of hematological parameters, the COL group showed statistically significant (*p* < 0.05) increases in monocyte, eosinophil, and basophil counts, whereas hemoglobin concentration decreased significantly (*p* < 0.01) in both groups. Furthermore, both groups demonstrated significant (*p* < 0.05) reductions in systolic and diastolic blood pressure, as well as improvement in physical performance in the TUG test, but no changes in handgrip strength. With regard to cognitive outcomes, COL supplementation significantly (*p* < 0.05) improved verbal memory, as reflected by the RAVLT score (39.88 ± 9.79 vs. 43.65 ± 12.05), whereas no significant changes were observed in the remaining cognitive tests. A significant (*p* < 0.05) time effect (pre- vs. post-intervention) was observed only for BMI, systolic blood pressure, and TUG performance, whereas a significant (*p* < 0.05) group effect was found only for hip circumference. The study showed that 12 weeks of supplementation with COL-enriched skim milk in older adults was associated with favorable metabolic effects (reductions in BMI, blood pressure, and LDL-C), cognitive benefits (improved verbal memory), and physical benefits (better TUG performance). Regular consumption of skim milk alone (PLA) also produced beneficial changes in body weight, blood pressure, and lower-limb functional performance, but did not improve verbal memory or LDL-C and, in fact, was associated with a reduction in HDL-C. However, the study did not demonstrate a significant group × time interaction for the assessed parameters, suggesting that COL was not clearly superior to PLA. According to the authors, regular consumption of milk, particularly skim milk enriched with bovine colostrum, may confer health benefits and support healthy aging in older adults.

## 4. Discussion

Based on the studies presented, it is difficult to draw unequivocal conclusions about the effects of bovine colostrum (COL) on body fat content and the blood lipid profile, as the studies were highly heterogeneous across multiple respects. The final number of participants varied considerably across studies (from 8 to 80), as did the number of experimental groups (from 1 to 4), and 3 studies did not include a placebo (PLA) group. Biological sex was not taken into account in all studies, and in two studies, only men were included. As indicated by the literature, there are important sex-related differences in fat deposition and reduction, as well as in lipid metabolism. These differences involve, among other factors, the distribution and accumulation of adipose tissue. In women, estrogen increases the expression of estrogen receptor α (ERα) in subcutaneous adipocytes, thereby promoting the differentiation of preadipocytes into subcutaneous fat. In men, by contrast, testosterone, together with lower estrogen concentrations, favors the accumulation of visceral fat, both through increased cortisol sensitivity and higher glucocorticoid receptor expression in this tissue [44,45]. Moreover, estrogen and testosterone regulate several pathways involved in lipid metabolism. One of these is hormone-sensitive lipase (HSL), which is responsible for the breakdown of triacylglycerols in adipocytes and appears to be more active in women. These hormones also modulate lipoprotein lipase (LPL), an enzyme that influences both the location and rate of fat deposition. In women, LPL activity predominates in subcutaneous adipose tissue, whereas in men it is more pronounced in visceral adipose tissue. Furthermore, de novo lipogenesis (DNL) occurs more intensively in the liver and adipocytes of men under the influence of androgens [46].

Another factor to consider is that ethnic background was reported in only three publications. As is well known, COL contains lactose. Therefore, in individuals with primary hypolactasia, a condition particularly prevalent among populations from East Asia, West Africa, and Native American groups, its digestion may be impaired. This, in turn, may reduce the absorption of its bioactive constituents [47]. In addition, participants’ geographical origin may influence the composition of the gut microbiota, which, in turn, affects the utilization of, among other components, lipids and oligosaccharides present in COL [48]. Within the intestine, bacterial lipases produced by genera such as *Bifidobacterium* and *Lactobacillus* hydrolyze triacylglycerols (TG) into glycerol and free fatty acids (FFA). These compounds, along with oligosaccharides, undergo fermentation, producing short-chain fatty acids (SCFAs) such as acetate, propionate, and butyrate. These fatty acids are absorbed through the intestinal epithelium into the portal circulation and subsequently transported to the liver. In the liver, acetate is utilized for the synthesis of cholesterol (cholesterogenesis), glucose (gluconeogenesis), and fatty acids (de novo lipogenesis). Propionate is converted primarily into glucose via gluconeogenesis, whereas butyrate contributes to triacylglycerol synthesis [49]. Through these mechanisms, COL consumption may influence both the blood lipid profile and adipose tissue accumulation. However, the clinical studies included in this systematic review did not consider the role of the gut microbiota in this context. It should also be emphasized that any effect associated with increased SCFA production, which might theoretically support lipolysis, is likely to depend on the individual composition of the gut microbiota.

The COL supplements used across the studies varied considerably in formulation. In some studies, the intervention consisted of liquid, pure, non-standardized colostrum, whereas in others it involved concentrated powder or tablet formulations, spray-dried commercial products containing >1%, 25%, or 30% IgG, hyperimmune colostrum, or skim milk enriched with colostrum. Fresh liquid COL is regarded as the gold standard in clinical research, as it exhibits the most potent immunomodulatory and anabolic activity; however, its shelf life is short, and it must be consumed within 24–48 h. By contrast, COL processed using technological methods such as freeze-drying, lyophilization, or spray-drying contains somewhat lower levels of biologically active constituents but can be stored for up to 2 years at room temperature, provided it is kept in airtight packaging and protected from moisture. Owing to its greater economic efficiency, this form is the one most commonly used in the commercial production of dietary supplements [50,51].

In our view, the use of such diverse COL formulations across the studies reviewed may have contributed to differences in their observed biological effects. Moreover, in the case of the multicomponent supplement RiteStart^®^, which contained COL in addition to various plant-derived ingredients, it is difficult to attribute the observed effects solely to colostrum. Importantly, not all studies reported whether the COL preparation had been subjected to analytical verification, nor did they consistently provide data on its energy and nutritional value; in some cases, the authors appeared to rely on manufacturer-provided information, although this was not always clearly stated. It is also noteworthy that COL was administered in markedly different doses and at different frequencies throughout the day: 5 g twice daily, 15 g of powder twice daily, 20 g once daily, 25 g once daily, 60 g once daily, 500 mg once daily, 600 mg three times daily, or as 500 cm^3^ of liquid or powdered preparation once daily. These differences may likewise have influenced the metabolic outcomes observed. Different vehicles were also used for reconstitution, including water, skim milk, and juice, which may have altered the total energy and nutritional value of the administered supplement. In addition, COL was consumed at different times of day: only in the morning; at a time convenient for the participant; after breakfast in the morning; morning and evening; after training on exercise days; 30 min before meals; or with meals (as in patients with short bowel syndrome). According to the literature, morning consumption in the fasting state may promote more efficient absorption of immunoglobulins, lactoferrin, and IGF-1, as higher serum concentrations of these components have been observed within several hours after ingestion. In contrast, evening consumption of COL (before sleep) may be more beneficial for muscle recovery and intestinal barrier restoration after intense exercise, when the body is in a more catabolic state and nocturnal protein synthesis occurs [52].

In the clinical studies reviewed, the findings were compared with those obtained in PLA groups; however, these comparator preparations did not consist of a single, uniform protein source. Most commonly, the PLA group received whey protein, as its protein content is broadly comparable to that of COL. Nevertheless, other combinations of animal- and plant-derived proteins were also used, including whey plus casein, whey protein isolate combined with soy protein isolate and rice protein, skim milk, and pasteurized milk powder. In one study, no information was provided on the composition of the PLA preparation. This should be considered when interpreting the findings. The significant differences observed between groups in the analyzed parameters may therefore have been influenced, at least in part, by the distinct biological properties of the comparator preparations. The absence of between-group differences may likewise be explained by this factor. As indicated in the literature, each of these protein sources has a different bioactive profile, which translates into distinct metabolic, immunological, and anabolic effects. Plant protein isolates constitute a valuable source of high-quality protein and are generally better tolerated by individuals with cow’s milk protein allergy; however, they lack the characteristic bioactive compounds present in COL, such as immunoglobulins, lactoferrin, and growth factors including IGF-1 and TGF-β. The combined use of diverse milk-derived protein sources (e.g., whey, casein, and colostrum) may be justified in studies with athletes, who have an increased requirement for high-quality protein [43,53,54].

Further limitations of the available studies include the variable duration of COL administration (4–12 weeks), whereas a meaningful reduction in body fat would likely require a longer intervention period (≥6 months) and/or the implementation of an energy deficit, together with careful control of the energy and nutritional value of the diet. In the studies analyzed, participants’ diets were not consistently monitored; when dietary intake was assessed, different methods were used to estimate macro- and micronutrient consumption, including repeated 24-h dietary recalls and 3-day food records collected before and after supplementation. Dietary energy intake was not restricted, and COL itself provides additional calories (approximately 80 kcal per 20 g), which may have attenuated any potential lipolytic effect on adipose tissue. Although COL contains IGF-1 and lactoferrin, these components do not directly stimulate lipolysis; rather, they promote protein synthesis and muscle accretion, which may explain why the main effect observed in some studies was an increase in lean body mass (LBM), likely in conjunction with physical activity, particularly resistance training.

Although COL contains lower levels of aflatoxin M1, pathogenic bacteria, other microorganisms, antibiotics, stimulants, and other undesirable substances than many commercially available dairy products, it is generally considered safe for use [4]. Given the importance of monitoring adverse events during dietary supplement use, we conducted a thorough re-evaluation of all 13 included studies to extract all available data on adverse events (AEs), monitoring methods, and authors’ conclusions. These data are presented in Supplementary Table S5.

Five studies used rigorous safety-monitoring methods, including dedicated adverse event forms, weekly contact with nurses, and investigator-guided causality assessments, as reported by Kerksick et al. [38], Duff et al. [39], Dukaew et al. [31], and Lund et al. [33]. In contrast, several studies in athletes relied primarily on self-reported symptoms or adherence monitoring, while others did not explicitly report safety outcomes.

Despite variation in adverse event reporting, the available data indicate that bovine colostrum is generally safe and well tolerated in healthy adults and athletes at doses of 20–60 g/day. The most commonly reported adverse events were mild gastrointestinal symptoms, including bloating, diarrhea, and nausea, which usually resolved spontaneously. Discontinuation due to intolerance was rare (<5% of participants) and occurred with similar frequency in the colostrum and placebo groups in most studies. This suggests that these symptoms may be related to high protein intake rather than to colostrum toxicity. However, the effects of colostrum in patients with intestinal dysfunction, particularly those with short bowel syndrome, warrant careful consideration. Nausea and vomiting were more common in these patients than in controls and led to discontinuation of the intervention in some cases. These findings indicate that, although colostrum appears safe for the general healthy population, caution is required when using it in specific clinical settings. Because some studies did not clearly report safety outcomes, standardized safety reporting should be incorporated into future trials to ensure a comprehensive assessment of the risk–benefit profile of colostrum supplementation. This limitation should be considered when interpreting the available evidence and may represent an important factor restricting the broader application of COL supplementation.

In our review, a systematic analysis of dose–response relationships was not possible due to heterogeneity in study designs, populations, and colostrum formulations, including differences in IgG content and processing methods. However, a qualitative synthesis of the 13 included studies suggests a possible threshold effect and indicates a potentially optimal dosage range for achieving specific outcomes, particularly reductions in body fat and improvements in blood lipid profiles. Based on the available evidence, lower doses (<10 g/day) or formulations with substantial variability often produced null findings, suggesting that a minimum efficacy threshold may be required to activate colostrum-related anabolic and metabolic pathways, potentially involving IGF-1 or leptin signaling. Accordingly, the minimum effective threshold appears to be approximately 20 g/day, providing approximately 150 mg of IgG, which may be sufficient to induce measurable physiological changes in healthy adults and athletes. However, above this threshold (up to 60 g/day), there is currently no clear evidence of a dose-dependent increase in benefits for body composition or blood lipid profiles. Therefore, based on the available qualitative evidence, we propose that a dose of 20–25 g/day may represent a practical and potentially optimal range for maximizing the effects of bovine colostrum on body composition, metabolic health, and athletic performance.

Among the clinical studies reviewed, only two [39,40] demonstrated a significant effect of COL on body fat reduction, both conducted in older adults. In addition to the limitations discussed above, the lack of significant differences in the remaining studies may also be attributable to the methods used to assess body fat content, namely skinfold thickness (SF), dual-energy X-ray absorptiometry (DXA), and bioelectrical impedance analysis (BIA). Although the SF method is technically simple and inexpensive, studies published over the past five years indicate that estimates of body fat content obtained with this method exhibit the greatest systematic error compared with gold-standard techniques such as DXA, BIA, or magnetic resonance imaging (MRI). The accuracy of SF measurements depends heavily on the examiner’s expertise, the anatomical sites selected for measurement, the precision of the equipment, and the interpretation method used, including older regression equations that may not be suitable for more diverse populations. Previous studies [55,56,57,58] have shown that the SF method tends to overestimate body fat at lower values and underestimate it at higher values. It may also underestimate body fat in older adults. In addition, it shows the weakest correlation with visceral adipose tissue, which is a key parameter in the assessment of metabolic health. Furthermore, compared with the more sensitive DXA method, it may be insufficiently sensitive to detect subtle changes in body composition.

According to the literature [49,59,60], the bioactive components of COL, including IGF-1, lactoferrin, TGF-β, PDGF, and EGF, may have beneficial effects on lipid profiles. Several mechanisms may underlie these effects. These include reduced cholesterol absorption, modulation of the gut microbiota, attenuation of inflammation, and improved insulin sensitivity. In addition, lactoferrin may affect the expression of lipid-related genes, while other components may help limit hepatic lipogenesis. Regarding the effects of COL on the lipid profile, beneficial effects were observed primarily in individuals with pre-existing metabolic disturbances, such as type 2 diabetes mellitus, or in older adults [28,40]. COL supplementation significantly reduced total cholesterol and LDL-C levels in diabetic and older adult populations. The authors of these studies attributed these effects to the presence of leptin and IGF-1 in COL, which may enhance insulin sensitivity and promote fatty acid oxidation in skeletal muscle, thereby reducing circulating triglyceride and cholesterol concentrations. Furthermore, Mizrahi et al. [41] observed that hyperimmune COL (Imm124-E) improved lipid profiles in patients with nonalcoholic steatohepatitis (NASH) by increasing adiponectin levels and modulating regulatory T cells (Tregs), thereby attenuating the chronic low-grade inflammation associated with dyslipidemia. In such metabolic disorders, the primary therapeutic effect appears to involve restoring glucose homeostasis and reducing insulin resistance, ultimately leading to favorable changes in lipid metabolism. In contrast, under conditions of immunological or exercise-induced stress, changes in blood lipid profiles may be less pronounced. Al-Nimer et al. [34] suggested that this may result from the prioritization of energy mobilization over lipid regulation. The authors proposed that the observed increase in stress-induced glycemia, in the absence of significant changes in triglyceride concentrations or the triglyceride–glucose (TyG) index, reflected COL’s role in supporting glucose delivery to skeletal muscle as a primary energy substrate during exercise while simultaneously stabilizing hemodynamic responses. According to their interpretation, the absence of lipid-lowering effects in healthy individuals may be explained by increased fatty acid utilization for energy production under physiological stress. In this context, COL appears to support metabolic and immune homeostasis rather than directly modulating lipid metabolism, distinguishing its mechanism of action from that observed in pathological states such as type 2 diabetes.

Similarly, Han et al. [29] reported that although COL supplementation improved immune markers, including secretory immunoglobulin A (sIgA), and increased blood vitamin concentrations, it did not significantly affect TC, HDL-C, or LDL-C levels in healthy adults. These findings suggest that, in the absence of underlying metabolic dysfunction, lipid-lowering pathways potentially mediated by IGF-1 may not be activated to the same extent.

Interestingly, colorectal cancer (CRC) has also been associated with abnormalities in blood lipid profiles [61,62,63]. Clinical interventional studies investigating colostrum, particularly its lactoferrin fraction, as an adjunctive therapy in patients with inflammatory bowel disease and early colonic lesions (e.g., polyps) have primarily focused on preventing progression of precancerous lesions and reducing intestinal inflammation. Although substantial evidence from in vitro and in vivo studies suggests the anticancer potential of colostrum, clinical studies in humans have mainly evaluated its supportive role in reducing postoperative complications, inflammation, and oxidative stress, and in promoting intestinal mucosal regeneration [64,65,66,67,68,69]. Clinical trials in CRC populations have primarily assessed the effects of COL on intestinal barrier integrity, immune modulation, and reduction in adverse events; however, none have evaluated changes in blood lipid profiles. This research gap regarding the effects of dietary COL supplementation on lipid metabolism in CRC patients may partly stem from theoretical concerns related to the high IGF-1 content of colostrum, given the established association between IGF-1 signaling and cancer cell proliferation [70,71]. Consequently, further preclinical and clinical studies are warranted to determine whether modified colostrum preparations, for example with reduced IGF-1 content, could safely improve dyslipidemia without compromising oncological safety.

Interpretation of null findings in the present review should also be approached with caution, particularly in relation to the study by Lund et al. [33], which included only eight participants. Such a small sample size substantially limits statistical power and reduces the likelihood of detecting anything other than large intervention effects. Therefore, the absence of statistically significant effects of COL supplementation on body fat content in this study should not be interpreted as evidence of ineffectiveness [72,73], but rather as an inconclusive finding with a high risk of Type II error. In accordance with PRISMA recommendations, this study was assessed using the RoB 2 tool and was rated as having a high risk of bias due to imprecision (Table 1). Accordingly, adequately powered randomized controlled trials are needed to provide more robust evidence regarding the effects of COL supplementation on body composition and metabolic outcomes.

The credibility of the available evidence should also be interpreted with caution, as one study included participants who were employees, family members, or acquaintances of employees of the company sponsoring the research, which may indicate a potential source of bias in favor of the tested product. On the other hand, it is noteworthy that the studies were conducted in a wide range of countries, including Australia, Canada, Denmark, Iraq, Israel, Malaysia, the Netherlands, Poland, Republic of Korea, Thailand, and the United States, which reflects broad scientific interest in COL supplementation across different regions of the world.

It should be emphasized that the interpretation of the findings of the present review must take into account the methodological limitations of the studies included in the analysis. Among the randomized trials, most were judged to have either some concerns or a high risk of bias, mainly due to insufficient reporting of randomization procedures and allocation concealment, attrition after randomization, and additional limitations inherent to crossover designs. Furthermore, all non-randomized studies were assessed as being at critical risk of bias, primarily because of the absence of a concurrent control group and the high likelihood of confounding. Consequently, the available evidence suggesting beneficial effects of COL on body fat content and lipid profile should be interpreted with caution, as it remains preliminary and insufficient to support strong clinical conclusions.

The GRADE assessment showed that the certainty of the evidence for the effects of bovine colostrum supplementation on body fat reduction and improvement in the blood lipid profile was predominantly very low. According to the GRADE framework, very low certainty means that the true effect may be substantially different from the observed estimates. Therefore, the findings of this review should be interpreted with caution and considered hypothesis-generating rather than confirmatory.

The certainty of evidence was downgraded mainly due to small sample sizes, incomplete reporting of randomization procedures, attrition, open-label or uncontrolled study designs, and limitations related to crossover trials. Additional concerns resulted from substantial heterogeneity in study populations, supplementation dose and duration, comparator type, and co-interventions. In several studies, bovine colostrum was administered as part of multi-ingredient preparations, which made it difficult to isolate its independent effect.

Another important limitation was the variability in body composition assessment methods, including DXA, BIA, and skinfold thickness measurements. Moreover, in several studies, favorable changes in body fat or lipid parameters were observed only within groups and were not confirmed by significant between-group differences.

In line with Cochrane/GRADE guidance, very low certainty does not exclude a potential biological effect of bovine colostrum, but indicates that the current evidence is insufficient to support firm conclusions. Therefore, future randomized, double-blind, placebo-controlled trials with larger samples, standardized bovine colostrum doses, longer intervention periods, controlled diet, and validated body composition methods, preferably DXA, are needed. At present, bovine colostrum supplementation cannot be recommended as an evidence-based strategy for reducing body fat or improving lipid profile in adults.

In summary, although in recent years increasing attention has been paid to the numerous health benefits potentially associated with COL supplementation in both healthy and diseased populations, and to emerging trends in this field [60,74], it is equally justified to focus on lipid metabolism and the role of the gut–adipose tissue axis in both adults and older individuals.

## 5. Conclusions

Based on the clinical studies reviewed, the evidence supporting the effect of bovine colostrum (COL) supplementation on body fat reduction and improvements in the blood lipid profile remains limited. A significant reduction in body fat content was reported in only two studies, both conducted in older adults, following supplementation with either 60 g/day for 8 weeks or 10 g/day for 12 weeks in combination with plant protein isolates. Similarly, only two studies demonstrated significant favorable changes in the blood lipid profile. In these studies, COL supplementation at 10 g/day for 4 weeks in individuals with type 2 diabetes mellitus and at 30 g/day for 12 weeks in older adults was associated with reductions in total cholesterol (TC), LDL-C, and triacylglycerol (TG) concentrations.

However, the available evidence is characterized by substantial methodological heterogeneity, including differences in study populations, intervention duration, COL dose, comparator formulations, and outcome assessment methods. These limitations preclude firm conclusions regarding the independent effects of COL on adiposity and lipid metabolism.

Well-designed randomized, double-blind, controlled trials are therefore warranted, particularly in overweight and obese individuals across different age groups. Future studies should ideally extend beyond 6 months, incorporate a controlled energy deficit (e.g., −500 kcal/day), apply a standardized COL dose (20–25 g/day), and include four intervention arms: (1) placebo + normocaloric diet, (2) COL + normocaloric diet, (3) placebo + energy-restricted diet, and (4) COL + energy-restricted diet. In addition to standard anthropometric outcomes, such trials should include assessments of blood biomarkers related to carbohydrate and lipid metabolism, body composition by DXA before and after the intervention, and analyses of the gut microbiota and the fecal short-chain fatty acid (SCFA) profile.

Such an approach would help clarify whether any observed reduction in body fat and improvement in the blood lipid profile can be attributed to COL supplementation per se, or whether these effects occur only when COL is combined with an energy-restricted dietary intervention.

## Figures and Tables

**Table 4:** Summary of Findings (SoF) according to the GRADE approach.

Outcome	Participants (Studies)	Summary of Effect	Certainty of Evidence
Body fat/fat mass/% body fat	257 (8 studies)	Most studies showed no significant advantage of COL over placebo or protein control for total fat mass or % body fat.	Very low
Skinfolds/adiposity proxy	56 (2 studies)	No consistent reduction in skinfold thickness was observed compared with the control.	Very low
Regional fat mass	80 (1 study)	One study showed a small reduction in lower-limb fat percentage; however, evidence was limited to a single trial.	Very low
Body weight/BMI/anthropometric parameters	421 (11 studies)	Findings were inconsistent and cannot be clearly attributed to bovine colostrum supplementation.	Very low
Total cholesterol (TC)	91 (4 studies)	Some studies reported reductions in TC, but findings were inconsistent across trials.	Very low
LDL-C	75 (3 studies)	Possible reductions in LDL-C were observed in selected studies; however, the evidence was inconsistent and imprecise.	Very low
HDL-C	65 (2 studies)	No reliable evidence of a beneficial improvement in HDL-C was identified.	Very low
Triglycerides (TG)	81 (3 studies)	One study demonstrated TG reduction, whereas the remaining studies did not.	Very low
TyGI/triglyceride-glucose index	50 (1 study)	One study assessed TyGI rather than the classical lipid profile, limiting comparability.	Very low

Explanations: Downgraded for serious risk of bias due to incomplete reporting of randomization/allocation concealment, attrition, crossover-specific limitations, and inclusion of open-label or multi-ingredient interventions. Downgraded for inconsistency because findings differed substantially across studies and populations. Downgraded for indirectness due to heterogeneous participant characteristics, supplementation protocols, comparators, co-interventions, and body composition assessment methods. Downgraded for imprecision because of small sample sizes and limited numbers of studies per outcome. Downgraded for suspected publication bias because relatively few studies were available and several interventions were industry supported or supplement provided.

**Table 5:** Characteristics of clinical studies conducted in adult humans regarding the impact of colostrum bovinum (COL) on the body fat content (*n* = 9).

Participants	Group	Duration	Intervention	Analysis	Results	References
Adults; N = 22; age 18–35 years (mean 24 years); men (*n* = 14), women (*n* = 8); ethnicity: no data; country: USA	(1) COL (*n* = 9) (2) PLA (*n* = 13)	8 weeks; aerobic training + heavy resistance training (3/week)	(1) COL 20 g (2) Whey 20 g, isocaloric, dissolved in water	Body composition (DXA); exercise performance (treadmill time to exhaustion; one repetition maximum (1RM) bench press; submaximal bench press endurance); energy and macronutrient intake (24 h dietary recall)	In the PLA group, BW ↑ (*p* < 0.05; 2.11 kg). In the COL group, BW ↑ (*p* < 0.05) and LBM ↑ (1.49 kg). No between-group differences were observed in FM. COL combined with training may promote greater gains in LBM than isocaloric whey protein. No significant differences were observed in strength or endurance outcomes between whey and placebo.	[36]
Adults; cyclists; N = 28; age 30 ± 10 years; men; BW 74 ± 21 kg; VO_2_max 61 ± 9 mL/kg/min; Σ7 skinfolds 67 ± 48 mm; ethnicity: no data; country: Australia	(1) PLA (*n* = 10) (2) COL (*n* = 9) (3) COL + WPC (*n* = 9)	8 weeks	(1) PLA 60 g + WPC (2) COL 60 g/day (3) COL 20 g/day + WPC 40 g/day; morning: 20 g COL; evening: 40 g COL; dissolved in skim milk	Measure One: 2 tests of VO_2_max separated by 20 min; Measure Two: 2 h cycling at 65% VO_2_max followed by an IGF-1 questionnaire; energy and macronutrient intake	No significant between-group differences were found in Measure One. IGF-1, blood variables, nutrient intake, and fluid intake were unchanged. In Measure Two (time trial), pre- to post-supplementation performance improved significantly (*p* < 0.05): COL ↓ 158 s; COL + WPC ↓ 134 s; PLA ↓ 58 s. Changes in Σ7 skinfolds in the COL group were not statistically significant.	[32]
Adults; hockey players; N = 28; men (*n* = 14), women (*n* = 14); age 18–27 years; ethnicity: no data; country: Netherlands	(1) COL (males = 7; females = 7) (2) PLA (males = 7; females = 7)	8 weeks; 4 training sessions/week	(1) COL 60 g/day (2) Whey protein 60 g/day (20 g morning; 40 g evening)	Anthropometric measurements; body composition (skinfolds); sprint; suicide run; vertical jump	In the PLA group, BW ↑ (*p* < 0.01), BF% ↓ by 0.1 ± 0.3 kg, and LBM ↑ by 1.2 ± 0.3 kg. In women, differences in FFM between PLA and COL were not significant. Sprint performance improved numerically in the COL group vs. PLA (−0.64 ± 0.09% vs. −0.33 ± 0.09%), although without statistical significance. No significant effects were observed for shuttle run, suicide run, or vertical jump, either within or between groups.	[37]
Adults; resistance-trained; N = 49; men (*n* = 36), women (*n* = 13); age 18–45 years; ethnicity: no data; country: USA	(1) PRO (2) PRO/COL (3) PRO/CR (4) COL/CR	12 weeks; body resistance training program (4/week)	(1) PRO (casein/whey 60 g/day) (2) PRO/COL (3) PRO/CR (4) COL/CR; COL dissolved in water/juice/milk; CR loading dose 20 g/day for 5 days, then maintenance dose 5 g/day	Body composition (BIA, DXA); maximal strength (one repetition maximum 1RM bench press and leg press); endurance	No between-group differences were observed in energy intake or dietary composition. Resistance training increased 1RM strength, muscular endurance, sprint capacity, and FFM in all groups. Compared with PRO, PRO/CR and COL/CR elicited greater increases in DXA-derived FFM during training (*p* < 0.05). The addition of CR to either PRO or COL resulted in slightly greater FFM gains over 12 weeks. However, strength and anaerobic capacity improved irrespective of supplement type. No independent ergogenic effect of COL was confirmed.	[38]
Adults; healthy; N = 50 (final *n* = 13); men (*n* = 7), women (*n* = 6); age 18–45 years; ethnicity: 1 Asian, 8 Hispanic, 4 White; country: USA	(1) RiteStart^®^ (2) COL + vitamins, minerals, botanical extracts, enzymes, omega-3 fatty acids	12 weeks	RiteStart^®^ 5 g twice daily (morning and evening)	Anthropometric measurements; body composition; blood biomarkers; salivary IgA	No significant effects of supplementation were observed on body composition, including FM, FFM, TBW, or BF% (*p* > 0.01).	[29]
Adults; endurance-trained; N = 58 (final *n* = 28); men; age 31.1 ± 10.2 years; ethnicity: no data; country: Poland	(1) COL (*n* = 13) (2) PLA (*n* = 15)	28 weeks; 12-week supplementation; 4-week washout; 3–5 training sessions/week	(1) COL (2 × 12.5 g/day) (2) PLA (2 × 12.5 g/day); powder dissolved in 250 cm^3^ water	Anthropometric measurements; body composition (BIA); incremental rowing test; serum testosterone	No significant between-group changes were observed in BW, TBW, FFM, or FM. Time to VT ↓ significantly only in the COL group vs. baseline, but not vs. PLA. No differences were found in exercise-induced responses between COL and PLA. The optimal COL dose may need to be individualized based on the training cycle.	[35]
Adults; elderly; N = 40; men (*n* = 15), women (*n* = 25); age ≥ 59 years; ethnicity: no data; country: Canada	(1) COL (*n* = 12 women, *n* = 7 men) (2) PLA (*n* = 13 women, *n* = 8 men)	8 weeks resistance training program (12 exercises, 3 sets of 8–12 reps, 3 days/week)	(1) COL 60 g/day (2) Whey protein 60 g/day; both provided as 3 × 20 g/day	Body composition (DXA); muscle thickness (ultrasound); muscle strength (1RM); cognitive function (TICS); creatinine and urinary K^+^; IGF-1 and CRP; 3-day food logs	Significant within-group increases were observed in BW, BMC, LBM, and biceps brachii thickness in both groups (*p* < 0.01). Significant between-group differences were detected only for walking distance. Total body K^+^ ↑ in the COL group. No between-group differences were found for IGF-1 or CRP. COL combined with resistance training improved leg press performance in older adults; however, the benefits over PLA were limited. No supplement group × sex × time interactions were observed.	[39]
Adults; elderly; N = 94 (final *n* = 80); men (*n* = 34), women (*n* = 46); age 55–70 years; ethnicity: no data; country: Thailand	(1) COL (*n* = 40; men = 18, women = 22) (2) PLA (*n* = 40; men = 16, women = 22)	12 weeks	(1) COL 10 g (2) PLA; twice daily (morning and evening), dissolved in 180 cm^3^ water	Whey-derived immunological markers; cytokines; multidimensional body composition assessment; anthropometric parameters; bone metabolism markers; cognitive function (MoCA)	After 12 weeks, COL increased serum IgG and IGF-1 and decreased serum osteocalcin relative to baseline, with some sex-related variation. No significant changes were observed in tumor markers, muscle function, or cognitive outcomes.	[31]
Adults with SBS; N = 12 (final *n* = 8); men (*n* = 7), women (*n* = 5); age 55.7 ± 10.7 years; ethnicity: no data; country: Denmark	(1) COL (2) PLA	4-week supplementation; 4-week washout; habitual diet	(1) COL 250 cm^3^/day (2) PLA 250 cm^3^/day, twice daily (morning and evening)	Physical examination; fluid and electrolyte balance; nutrient balance studies; body composition; urinary excretion; plasma GLP-2; maximal handgrip strength; lung function	Both COL and PLA increased energy and protein absorption; however, BW ↑ and LBM ↑ were observed only after PLA. No differences between interventions were found in body composition, physical function, or metabolic parameters.	[33]

Explanations are in the Abbreviations section.

**Table 6:** Characteristics of clinical studies conducted in adult humans regarding the impact of colostrum bovinum (COL) on the blood lipid profile (*n* = 6).

Participants	Group	Duration	Intervention	Analysis	Results	References
Adults, type 2 diabetes; N = 16 Age: 35–65 y Sex: men (*n* = 8), women (*n* = 8) Ethnicity: Asian Country: South Korea	COL	4 weeks	(1) COL (5 g/day), administered twice daily (morning and evening); powder	Blood analysis: glucose (fasting; 2 h and 8 h postprandially), TG, TC, β-hydroxybutyric acid	glucose ↓, TC↓, and TG↓ were significant. No significant changes were observed in β-hydroxybutyric acid.	[40]
Adults with type 2 diabetes and NASH; N = 10 Age: 18–60 y Sex: men, women Ethnicity: no data Country: Israel	Imm124-E with COL	30 days; 60-day post-intervention observation	600 mg, three times daily (1800 mg/day)	Physical examination; laboratory tests: complete blood counts (CBC), sedimentation rate (ESR), liver enzymes, lipid profile, CRP, IL-6, GLP-1, HbA1c, serum insulin, OGTT, HOMA-IR	HbA1c ↓ significantly (*p* < 0.03; ~14.8%). Tregs (CD4^+^CD25^+^HLA-DR^+^) ↑ significantly (*p* = 0.002; 2.3% to 3.8%). No significant improvement was observed in glucose, insulin, OGTT, HOMA-IR, GLP-1, IL-6, adiponectin, ALT, AST, AP, γ-GT, TC, or LDL-C. No effect on body mass was observed.	[41]
Adults, healthy; N = 50 (final *n* = 13) Sex: men (*n* = 7), women (*n* = 6) Age: 18–45 y Ethnicity: 1 Asian, 8 Hispanic, 4 White Country: USA	RiteStart^®^ (COL + vitamins + minerals + botanical extracts + omega-3 fatty acids)	12 weeks	RiteStart^®^ 5 g, twice daily (morning and evening); powder	Anthropometric measurements; body composition; biomarkers in blood; sIgA in saliva	After supplementation, salivary IgA ↑ significantly, as did glucose, folic acid, and SHBG. RBC, MCV, and RDW ↓ significantly. No significant changes were observed in ALT, AST, GGT, hsCRP, iron, potassium, sodium, TG, TC, HDL-C, or LDL-C. No significant changes in body composition were found.	[29]
Adults, healthy, exercise-trained; N = 50 Sex: men (*n* = 50) Age: 18–25 y Ethnicity: no data Country: Iraq	(1) PLA (*n* = 24) (2) COL (*n* = 26)	8 weeks; resistance training, 2 h, 3 sessions/week	(1) PLA (500 mg/day) (2) COL (500 mg/day); single oral dose; nutraceutical pill	Anthropometric measurements; hemodynamic parameters (blood pressure, RPP); morphology and biochemical blood markers (FBG, HbA1c%, TG); stress hyperglycemia ratio (SHR); triglyceride–glucose index (TyGI)	No between-group differences were observed for BMI or WC. FBG remained unchanged in both groups; HbA1c and TyGI were unchanged in the PLA group. MCV ↓ significantly, and RPP ↑ significantly, in the PLA group. In the COL group, Hb ↑ and HbA1c ↑ significantly. The COL group also showed significantly higher stress hyperglycemia-related indices, including FBG, SHR, and TyGI.	[34]
Adults, older; N = 66 (final *n* = 52) Sex: men (*n* = 23), women (*n* = 29) Age: 50–69 y Ethnicity: Asian Country: Malaysia	(1) PLA (*n* = 26) (2) COL (*n* = 26)	12 weeks	(1) PLA (pasteurised milk powder) (2) COL (pasteurised milk powder providing 150 mg IgG in each sachet); 2 sachets twice daily; 15 g/day	Sociodemographic factors; anthropometric measurements; blood parameters (morphology, lipid profile, glucose); blood pressure; cognitive function (MMSE, RAVLT, Digit Span, Digit Symbol); physical fitness (TUG, grip strength); quality of life (WHOQOL-BREF)	In the COL group, TC ↓ and LDL-C ↓ significantly improved; monocytes, eosinophils, and basophils ↑; and verbal memory (RAVLT) improved. In the PLA group, waist circumference ↓ significantly and HDL-C ↓. In both groups, BMI ↓, hip circumference ↓, blood pressure ↓, and haemoglobin ↓ significantly; TUG performance improved.	[28]

Explanations are in the Abbreviations section.

## Data Availability

The bibliographic query in the Repository for Open Data https://doi.org/10.18150/KF1IKG.

## Supplementary Materials

The following supporting information can be downloaded at: https://www.mdpi.com/article/10.3390/nu18101579/s1, Table S1: GRADE evidence profile—Colostrum bovinum and body fat/lipid profile outcomes, Table S2: Controlled studies—body fat/adiposity/body composition, Table S3: Controlled studies—lipid profile, Table S4: Single-arm/open-label studies—not pooled quantitatively with randomized controlled trials, Table S5: Summary of Safety Profile and Adverse Events (AE) Across 13 Included Studies on Bovine Colostrum (COL) Supplementation on human body fat content and/or blood lipid profile.

## Abbreviations

The following abbreviations are used in this manuscript: 1RM, one-repetition maximum; ALT, alanine aminotransferase; AP, alkaline phosphatase; ASP, aspartate aminotransferase; BAT, brown adipose tissue; BF, Body fat; BIA, bioelectrical impedance analysis; BMC, bone mineral content; BMD, bone mineral density; BMI, body mass index; BW, body weight; CBC, complete blood counts; COL, colostrum; CR, creatine; CRP, *C*-reactive protein; CVD, cardiovascular diseases; DHEAS, dehydroepiandrosterone-sulfate; DNL, de novo lipogenesis; DXA, dual-energy X-ray absorptiometry; EGF, epidermal growth factor; ELISA, enzyme-linked immunosorbent assay; ESR, sedimentation rate; FGB, fasting blood glucose; FFA, free fatty acids; FFM, free fat mass; FM, fat mass; GH, Growth hormone; GGT (γ-GT), gamma-glutamyl transpeptidase; GLP-2, glucagon-like peptide; HB, haemoglobin; HbA1c, glycated haemoglobin; HDL-C, high-density lipoprotein; HOMA-IR, homeostatic model assessment of insulin resistance; HSL, hormone-sensitive lipase; IGF-1, insulin-like growth factor; IgG, immunoglobulin G; IL, interleukin; IRT, Incremental Rowing Test; LBM, bone-free lean body mass; LDL-C, low-density lipoprotein; LPL, lipoprotein lipase; LPS, lipopolysaccharides; LTM, lean tissue mass; MCV, mean cell volume; MMSE, Mini-Mental State Examination; MoCA, Thai Montreal Cognitive Assessment; NASH, nonalcoholic steatohepatitis; Ntx, cross-linked *N*-telopeptides of type I collagen; OGTT, oral glucose tolerance test; P1NP, procollagen type I *N*-terminal peptide; PDGF, platelet-derived growth factor; PLA, placebo; PRO, protein control; RBC, red blood cell count; RDW, red cell distribution width; RPP, rate pressure product; RAVLT, Rey Auditory Verbal Learning Test; SBS, short bowel syndrome; SCFA, short-chain fatty acids; SF, skinfold; sIgA, secretory immunoglobulin A; SGR, stress glucose ratio; SHBG, sex hormone-binding globulin; SHR, stress hyperglycemia ratio; TBW, total body water; TC, total cholesterol; TFAT, trunk fat; TFM, trunk fat mass; TG, triglycerides; TICS, Telephone Interview of Cognitive Status; TMSE, Thai Mental State Examination; TUG, Timed-Up-and-Go; TyGI, triglyceride-glucose index; VLDL-C, very-low-density lipoprotein cholesterol; VT, ventilatory threshold; WAT, white adipose tissue; WBC, white blood cells; WC, waist circumference; WHOQOL-BREF, World Health Organisation Quality of Life Abbreviated Version questionnaire; WPC, whey protein concentrate.
