# Supplementary material for: The Effects of Colostrum Bovinum Supplementation on Human Body Fat Content and/or Blood Lipid Profile: A Systematic Review of Clinical Trials
Source: Nutrients. 2026 May 15;18(10):1579. doi: 10.3390/nu18101579 (PMC13210282; doi:10.3390/nu18101579)
Supplement: Supplementary file 1 [file nutrients-18-01579-s001.zip › nutrients-4278847-supplementary.pdf]

## Supplementary Material 1

Supplementary **Table S1.** GRADE evidence profile — *Colostrum bovinum* and body fat/lipid profile outcomes

| Outcome | Studies reporting the outcome | No. of studies | Participants analyzed | Risk of bias | Inconsistency | Indirectness | Imprecision | Publication bias | Overall certainty | Justification |
|---|---|---|---|---|---|---|---|---|---|---|
| Body fat/fat mass / % body fat — direct body-composition outcomes | Antonio et al. (2001); Hofman et al. (2002); Kerksick et al. (2007); Han et al. (2023); Durkalec-Michalski et al. (2025); Duff et al. (2014); Dukaew et al. (2025); Lund et al. (2012) | 8 | 257 | Serious | Serious | Serious | Serious | Suspected | Very low | Most studies did not show a significant advantage of COL over placebo/protein control. Evidence was downgraded for methodological limitations, heterogeneous populations and interventions, and small samples. |
| Skinfolds as an adiposity proxy | Coombes et al. (2002); Hofman et al. (2002) | 2 | 56 | Serious | Not serious | Serious | Serious | Suspected | Very low | Skinfolds were used as an indirect measure of adiposity. No consistent reduction was demonstrated compared with the control. |
| Regional fat mass | Dukaew et al. (2025) | 1 | 80 | Serious | Not assessable | Serious | Serious | Suspected | Very low | One study reported a small reduction in lower-limb fat percentage; evidence is limited to a single trial and cannot support firm conclusions. |
| Body weight / BMI / anthropometric parameters | Antonio et al. (2001); Hofman et al. (2002); Kerksick et al. (2007); Han et al. (2023); Durkalec-Michalski et al. (2025); Duff et al. (2014); Dukaew et al. (2025); Lund et al. (2012); Mizrahi et al. (2012); Ooi et al. (2024); Al-Nimer et al. (2023) | 11 | 421 | Serious | Serious | Serious | Serious | Suspected | Very low | Anthropometric outcomes were inconsistent and cannot be attributed specifically to COL because body weight/BMI does not distinguish fat from lean mass, and were frequently affected by co-interventions. |
| Total cholesterol (TC) | Kim et al. (2009); Mizrahi et al. (2012); Han et al. (2023); Ooi et al. (2024) | 4 | 91 | Serious | Serious | Serious | Serious | Suspected | Very low | Some studies reported TC reductions, but results were inconsistent and included non-randomized/open-label or selected populations. |
| LDL-C | Mizrahi et al. (2012); Han et al. (2023); Ooi et al. (2024) | 3 | 75 | Serious | Serious | Serious | Serious | Suspected | Very low | Possible reductions in LDL-C were observed in selected studies, but the evidence was inconsistent, imprecise, and partly derived from non-randomized designs. |
| HDL-C | Han et al. (2023); Ooi et al. | 2 | 65 | Serious | Serious | Serious | Serious | Suspected | Very low | No reliable evidence of a |

| | | | | | | | | | | |
|---|---|---|---|---|---|---|---|---|---|---|
| | (2024) | | | | | | | | | beneficial improvement in HDL-C was identified. |
| Triglycerides (TG) — directly reported | Kim et al. (2009); Han et al. (2023); Ooi et al. (2024) | 3 | 81 | Serious | Serious | Serious | Serious | Suspected | Very low | One study reported TG reduction, whereas others did not confirm a consistent benefit. |
| TyGI / triglyceride-glucose index | Al-Nimer et al. (2023) | 1 | 50 | Serious | Not assessable | Serious | Serious | Suspected | Very low | TyGI was assessed instead of a classical lipid profile; comparability with lipid-profile outcomes is limited. |

**GRADE explanations.** Downgrading was applied for serious risk of bias, inconsistency, indirectness, imprecision, and suspected publication bias. Very low certainty indicates that the true effect may be substantially different from the observed estimate.

**Abbreviations:** COL, bovine colostrum; PLA, placebo/control; DXA, dual-energy X-ray absorptiometry; BIA, bioelectrical impedance analysis; TC, total cholesterol; LDL-C, low-density lipoprotein cholesterol; HDL-C, high-density lipoprotein cholesterol; TG, triglycerides; TyGI, triglyceride-glucose index; NR, not reported/extractable from available text; CI, confidence interval; SD, standard deviation; SEM, standard error of the mean; CR, creatine; PRO, protein control; WPC, whey protein concentrate.

Supplementary **Table S2.** Controlled studies — body fat/adiposity/body composition

| Outcome | Study | Time point | Mean COL | SD/SE/CI COL | n COL | Mean PLA/control | SD/SE/CI PLA | n PLA | Notes |
|---|---|---|---|---|---|---|---|---|---|
| Fat mass, kg | Antonio et al. (2001) | Baseline | 14.91 | 6.76 SD | 9 | 14.32 | 8.81 SD | 11 | DXA |
| Fat mass, kg | Antonio et al. (2001) | Post 8 weeks | 14.19 | 7.11 SD | 9 | 15.77 | 8.62 SD | 11 | No significant change in fat mass |
| Body fat, % | Antonio et al. (2001) | Baseline | 18.8 | 8.3 SD | 9 | 18.7 | 10.1 SD | 11 | DXA |
| Body fat, % | Antonio et al. (2001) | Post 8 weeks | 17.6 | 7.9 SD | 9 | 20.2 | 10.1 SD | 11 | No between-group difference |
| Sum of 7 skinfolds, mm | Coombes et al. (2002) | Baseline | 69 / 64 | 19 / 32 SD | 9 / 9 | 70 | 28 SD | 10 | COL 20 g / COL 60 g / PLA |
| Sum of 7 skinfolds, mm | Coombes et al. (2002) | Post 8 weeks | 64 / 58 | 18 / 20 SD | 9 / 9 | 68 | 22 SD | 10 | Skinfolds; not direct DXA fat mass |
| Fat mass change, kg | Hofman et al. (2002) | Change 0–8 weeks | −0.2 | 0.4 SEM | 15 | −0.2 | 0.4 SEM | 13 | No significant difference between COL and whey |
| Skinfolds change, mm | Hofman et al. (2002) | Change 0–8 weeks | −3.4 | 1.6 SEM | 15 | −3.9 | 1.8 SEM | 13 | No significant difference between groups |
| DXA body fat, % | Kerksick et al. (2007) | Baseline | 19.3 | 7.3 SD | 13 | 17.3 | 5.0 SD | 12 | PRO/COL vs PRO |
| DXA body fat, % | Kerksick et al. (2007) | Week 12 | 19.8 | 7.2 SD | 13 | 16.9 | 4.2 SD | 12 | No significant change in body fat |
| DXA body fat, % | Kerksick et al. (2007) | Baseline | 18.6 | 8.4 SD | 11 | 21.7 | 7.7 SD | 13 | COL/CR vs PRO/CR |
| DXA body fat, % | Kerksick et al. (2007) | Week 12 | 18.7 | 8.2 SD | 11 | 21.2 | 7.1 SD | 13 | COL with creatine; interpret separately |
| Fat mass, kg | Durkalec-Michalski et al. (2025) | Baseline | 15.1 | 6.3 SD | 28 crossover | 13.9 | 5.2 SD | 28 crossover | COLPRE vs PLAPRE |
| Fat mass, kg | Durkalec-Michalski et al. (2025) | Post 12 weeks | 14.8 | 6.7 SD | 28 crossover | 15.7 | 7.1 SD | 28 crossover | Crossover; no significant effect |
| Fat mass, % | Durkalec-Michalski et al. (2025) | Baseline | 17.9 | 5.7 SD | 28 crossover | 16.7 | 5.2 SD | 28 crossover | BIA |
| Fat mass, % | Durkalec-Michalski et al. (2025) | Post 12 weeks | 17.5 | 6.0 SD | 28 crossover | 18.5 | 7.1 SD | 28 crossover | No significant effect |
| Fat mass, kg | Duff et al. (2014) | Baseline | 27.5 | 13.0 SD | 19 | 25.0 | 9.3 SD | 18 | DXA |
| Fat mass, kg | Duff et al. (2014) | Post 8 weeks | 27.4 | 13.1 SD | 19 | 24.8 | 9.0 SD | 18 | No significant change in fat mass |
| Total tissue fat, % | Dukaew et al. (2025) | Mean difference at week 12 | −0.39 | 95% CI −1.04 to 0.26 | 40 | Reference | — | 40 | No significant difference |
| Total fat mass, g | Dukaew et al. (2025) | Mean difference at week 12 | −262.00 | 95% CI −748 to 225 | 40 | Reference | — | 40 | No significant difference |

| | | | | | | | | | |
|---|---|---|---|---|---|---|---|---|---|
| Legs tissue fat, % | Dukaew et al. (2025) | Mean difference at week 12 | −0.63 | 95% CI −1.20 to −0.06 | 40 | Reference | — | 40 | Significant difference favoring COL, $p = 0.0310$ |
| Total fat mass, kg | Lund et al. (2012) | Baseline | 16.45 | 9.9 SD | 8 crossover | 16.84 | 8.9 SD | 8 crossover | COL baseline vs control baseline |
| Total fat mass, kg | Lund et al. (2012) | Post 4 weeks | 16.83 | 9.6 SD | 8 crossover | 16.81 | 9.3 SD | 8 crossover | No superiority of COL |

Supplementary **Table S3.** Controlled studies — lipid profile

| Outcome | Study | Time point | Mean COL | SD COL | n COL | Mean PLA | SD PLA | n PLA | Notes |
|---|---|---|---|---|---|---|---|---|---|
| Total cholesterol, mmol/L | Ooi et al. (2024) | Baseline | 5.88 | 1.56 | 26 | 5.21 | 0.92 | 26 | COL vs skim-milk placebo |
| Total cholesterol, mmol/L | Ooi et al. (2024) | Post 12 weeks | 5.38 | 1.19 | 26 | 5.05 | 1.19 | 26 | Decrease in COL; no significant group × time interaction |
| HDL-C, mmol/L | Ooi et al. (2024) | Baseline | 1.53 | 0.49 | 26 | 1.42 | 0.32 | 26 | — |
| HDL-C, mmol/L | Ooi et al. (2024) | Post 12 weeks | 1.47 | 0.48 | 26 | 1.32 | 0.27 | 26 | HDL decreased in PLA |
| LDL-C, mmol/L | Ooi et al. (2024) | Baseline | 3.68 | 1.47 | 26 | 3.08 | 0.89 | 26 | — |
| LDL-C, mmol/L | Ooi et al. (2024) | Post 12 weeks | 3.28 | 1.14 | 26 | 3.06 | 0.79 | 26 | LDL decreased in COL |
| TG, mmol/L | Ooi et al. (2024) | Baseline | 1.46 | 0.75 | 26 | 1.58 | 0.60 | 26 | — |
| TG, mmol/L | Ooi et al. (2024) | Post 12 weeks | 1.39 | 0.89 | 26 | 1.48 | 0.78 | 26 | No significant TG change |

Supplementary **Table S4.** Single-arm / open-label studies — not pooled quantitatively with randomized controlled trials

| Outcome | Study | Time point | Mean baseline | SD baseline | n | Mean follow-up | SD follow-up | Notes |
|---|---|---|---|---|---|---|---|---|
| Total cholesterol, mg/dL | Han et al (2023) | Week 0 vs week 12 | 192.62 | 29.11 | 13 | 194.08 | 29.51 | No significant change |
| HDL-C, mg/dL | Han et al (2023) | Week 0 vs week 12 | 43.85 | 8.32 | 13 | 45.85 | 9.75 | No significant change |
| LDL-C, mg/dL | Han et al (2023) | Week 0 vs week 12 | 121.31 | 23.54 | 13 | 113.62 | 19.47 | No significant change |
| TG, mg/dL | Han et al (2023) | Week 0 vs week 12 | 160.85 | 78.33 | 13 | 177.62 | 110.40 | No significant change |
| Total cholesterol | Kim et al. (2009) | 4 weeks | NR | NR | 16 | NR | NR | The article reports a significant TC decrease; full numerical table values were not extractable from the available text |
| TG | Kim et al. (2009) | 4 weeks | NR | NR | 16 | NR | NR | The article reports a significant TG decrease; full numerical table values were not extractable from the available text |
| LDL-C / TC | Mizrahi et al. (2012) | 30 days | NR | NR | 10 | NR | NR | Open-label; lipid improvement reported in a subset; not suitable for classical RCT table |
| TyGI | Al-Nimer et al. (2023) | 8 weeks | NR | NR | 26 COL / 24 PLA | NR | NR | Reports TyGI and TG as part of the index, not a classical lipid profile |

Supplementary **Table S5.** Summary of Safety Profile and Adverse Events (AE) Across 13 Included Studies on Bovine Colostrum (COL) Supplementation on human body fat content and/or blood lipid profile

| Study | Population | Dose & Duration COL | Adverse Events (AE) Reported | Severity & Outcome | Notes on Tolerance & Compliance | Method of AE Assessment |
|---|---|---|---|---|---|---|
| Antonio et al. (2001) | Adults, active men & women, N=22 | COL 20 g<br>8 weeks | Not reported. | N/A | Two women dropped out for "unknown personal reasons" (not linked to AE). High compliance noted. | Self-reported logs; compliance monitoring via returned sachets. |
| Coombes et al. (2002) | Adults, competitive cyclists<br>N= 28 | 20 g or 60 g/day<br>8 weeks | Not reported. | N/A | 14 participants withdrew due to non-compliance (training/supplement adherence) but not explicitly linked to AE. | Daily training/diet diaries; monitoring of supplement compliance. |
| Hofman et al. (2002) | Adults, elite field hockey players<br>N=28 | 60 g/day<br>8 weeks | Not reported. | N/A | 7 participants dropped out (4 Whey, 3 COL). 1 person in the Whey group had "tolerance problems". | Return of unused sachets; monitoring of compliance. |
| Kerksick et al. (2007) | Adults, resistance-trained subjects<br>N=49 | 60 g/day<br>(COL or Blend)<br>12 weeks | Mild GI symptoms: Bloating, cramps, diarrhea reported in n<10 of participants across all groups (including placebo). | Mild;<br>did not compromise compliance. | 1 participant withdrew due to inability to consume supplement (GI issues). No severe AE. | Weekly contact with research nurse; self-reporting of side effects (bloating, cramps, diarrhea). |
| Han et al. (2023) | Adults,Athletes (Pilots)<br>N=50 (finally 13) | RiteStart<br>12 weeks | Not reported. | N/A | No mention of AE or dropouts related to supplement intolerance. | Not explicitly detailed in the article provided. |
| Durkalec-Michalski et al. (2025) | Adults, endurance athletes (Triathletes/Swimmers)<br>N=58 (finally 28) | 25 g/day<br>12 weeks | Not reported. | N/A | High dropout rate (30/58) noted, but primarily due to withdrawal during washout period or injuries, not supplement toxicity. | Monitoring of dropouts; standard safety checks (as implied by the crossover design). |

| | | | | | | |
|---|---|---|---|---|---|---|
| Duff et al. (2014) | Older adults<br><br>(50+ y)<br><br>N=40 | 60 g/day<br>8 weeks | GI Symptoms: 5 participants reported issues.<br>• Colostrum (2): Bloating, nausea, diarrhea, unsettled stomach ('mild', 'probable').<br>• Whey (3): GERD ('moderate'), nausea ('mild'). | Mild to Moderate.<br>• 2 participants in the Whey group discontinued due to "definite" relation to the supplement. | GI symptoms were slightly more frequent/severe in the Whey group than the COL group in this trial. | Adverse event forms; participant logs; investigator classification of causality ("definite", "probable"). |
| Dukaew et al. (2025) | Elderly (55–70 y)<br><br>N=94 (finally 80) | 100 g/day (50g x 2; 5g pure colostrum)<br>12 weeks | GI Symptoms: Mild diarrhea, bloating, constipation, flatulence, and nausea.<br>No significant difference between COL and PLA groups. | Mild; self-resolving (1–2 days). | Most events occurred early in supplementation.<br><br>All participants completed the study. No severe AE. | Self-reports by participants; investigator observations; physical exams & lab tests at baseline/end. |
| Lund et al. (2012) | Adults with Short Bowel Syndrome (SBS) patients<br><br>N= 12 (finally 8) | ~53 g protein/day (250ml colostrum x 2)<br><br>4 weeks | GI Symptoms: Nausea and vomiting.<br><br>2 patients discontinued due to intolerance to COL | Moderate; led to discontinuation<br>. | Colostrum was less tolerated than the milk/whey-based control in this specific clinical population. | 72-h balance studies; daily diaries; physical exams; monitoring of nausea/vomiting. |
| Kim et al. (2009) | Adults, T2 Diabetes patients<br><br>N=16 | 5 g/day (2x)<br>4 weeks | Not reported. | N/A | The study focused on lipid/glucose parameters; no safety data were provided. | Routine clinical monitoring (blood glucose, lipids). |
| Mizrahi et al. (2012) | Adults, NASH patients,<br><br>N=10 | Imm124-E with COL<br>30 days | Not reported. | N/A | Small pilot study; no safety data provided in the article. | Not explicitly detailed; pilot study design. |
| Al-Nimer et al. (2024) | Adults, exercise-trained healthy subjects<br><br>N= 50 | 500 mg g/day (COL)<br><br>8 weeks | Not reported. | N/A | The study focused on the Stress-Hyperglycemia Ratio and the TyG index. No adverse events or withdrawals due to intolerance were mentioned in the results. High compliance implied by completion of metabolic testing. | Not explicitly detailed; implied monitoring via completion of metabolic tests. |

| | | | | | | |
|---|---|---|---|---|---|---|
| Ooi et al. (2024) | Older adults (50–69 y)<br><br>N= 66 (finally 52) | 30 g/day (COL-enriched skim milk) 12 weeks | Not explicitly reported in the provided text. | N/A | No withdrawals due to AE mentioned. Study focused on metabolic outcomes. | Routine clinical monitoring; physical exams. |

AE: Adverse Events; COL: Colostrum; GI: Gastrointestinal; NASH: Non-alcoholic steatohepatitis; N/A - Not Applicable or Not Available; PLA: Placebo; SBS: Short Bowel Syndrome.
